# Capacity-Achieving Input Distributions of Additive Vector Gaussian Noise Channels: Even-Moment Constraints and Unbounded or Compact Support

**DOI:** 10.3390/e25081180

**Published:** 2023-08-08

**Authors:** Jonah Eisen, Ravi R. Mazumdar, Patrick Mitran

**Affiliations:** Department of Electrical & Computer Engineering, University of Waterloo, 200 University Ave W, Waterloo, ON N2L 3G1, Canada; mazum@uwaterloo.ca (R.R.M.); pmitran@uwaterloo.ca (P.M.)

**Keywords:** amplitude constraint, even-moment constraint, capacity-achieving distribution, Gaussian noise, spherically asymmetric channel

## Abstract

We investigate the support of a capacity-achieving input to a vector-valued Gaussian noise channel. The input is subjected to a radial even-moment constraint and is either allowed to take any value in $R^{n}$ or is restricted to a given compact subset of $R^{n}$. It is shown that the support of the capacity-achieving distribution is composed of a countable union of submanifolds, each with a dimension of n−1 or less. When the input is restricted to a compact subset of $R^{n}$, this union is finite. Finally, the support of the capacity-achieving distribution is shown to have Lebesgue measure 0 and to be nowhere dense in $R^{n}$.

## 1. Introduction

In this paper, we consider the support of the capacity-achieving input to a vector-valued channel that is subject to additive non-degenerate Gaussian noise. Vector-valued channels are used in a variety of applications, including the complex-valued inputs and outputs of quadrature channels, which have alternate representations as two-dimensional real vectors. Larger antenna arrays enable Multiple-Input Multiple-Output (MIMO) channels, which have inputs of n≥1 complex components. Additionally, noise with memory can be expressed by correlated noise components in a vector-valued channel.

Throughout the paper, the average input power is bounded to limit the consumption of environmental, battery, and monetary resources. Since the output of the amplifiers used in transmitters is severely distorted when the input is too large [1,2,3] and signals that are too small can be challenging to produce, it is also of practical interest to restrict the input to an arbitrary compact set.

There has been appreciable prior effort dedicated to understanding the capacity-achieving input to vector-valued channels subject to average power constraints and restrictions to compact sets. Nevertheless, there are significant technical challenges in working with vector-valued inputs. Therefore, much of the work to this point has been limited to either one-dimensional channels [4] or spherically symmetric channels [5,6,7,8], where the latter case ensures that the capacity-achieving distribution can be expressed as a univariate function of the radius. However, this restriction limits the scope of study to channels in which the input is constrained to a ball and the noise components are independent and identically distributed. In this paper, the only assumption made on the Gaussian noise distribution is that it is non-degenerate. We consider both cases, those in which inputs are restricted to arbitrary compact sets and those in which inputs are allowed to take any value in $R^{n}$.

The power of a vector-valued signal is equivalent to the second moment of its Euclidean norm. A constraint on the fourth moment then has the practical interpretation of limiting the second moment of the instantaneous power. Furthermore, imposing a moment constraint of order 2k ensures that the tails of the input distribution decay at least as quickly as a degree 2k monomial. Therefore, increasing the even-moment constraint penalizes large inputs without imposing a strict cutoff. This motivates us to generalize the average power constraint by limiting some even moment of the input’s Euclidean norm.

The results in this paper apply to any combination of input constraints described above, except for the special case where the input is allowed to take any value in $R^{n}$ and is subject to a second-moment constraint. This case reduces to a classical result in which the capacity-achieving distribution is known to be Gaussian. For all other cases, we show that the capacity-achieving distribution is contained in a countable union of *i*-dimensional submanifolds, where *i* ranges {0,…,n−1}. Furthermore, this union is finite when the input is restricted to a compact set. We then show that the support of the capacity-achieving distribution is a nowhere dense set with Lebesgue measure 0.

The paper is organized as follows. We first give a review of prior work in Section 2. Section 3.1, Section 3.2 and Section 3.3 provide intermediary results prior to the main results in Section 3.4. Section 4 concludes the paper.

## 2. Prior Work

Dating back to Shannon’s work in [9], much of the research on continuous channels has focused on average power (equivalently, second-moment) constraints on the input. A transmitter’s inability to produce arbitrarily large powers then led to the consideration of additional peak power constraints, modeled by restricting the input almost surely to compact sets.

The first major result on amplitude-constrained channels considers a scalar Additive White Gaussian Noise (AWGN) model, both with and without a variance constraint [4]. In each case, the support of the capacity-achieving distribution has a finite number of points.

The use of the Identity Theorem for functions of a single complex variable is key to the argument of [4] and many papers that follow. The theorem can be applied to any univariate analytic function that has an accumulation point of zeros. By contrast, the Identity Theorem in *n* complex dimensions requires an analytic function with an open set of zeros in $C^{n}$. Therefore, to apply the Identity Theorem directly for n>1, a random vector with support containing an open subset of $C^{n}$ must be considered. It was suspected by some authors that, since $R^{n}$ is not open in $C^{n}$, no topological assumption on the support of the capacity-achieving distribution would be sufficient for this purpose [5,10,11]. Therefore, many papers restrict their models to ones that maintain spherical symmetry so that the capacity-achieving distribution can be expressed as a one-dimensional function of radius (e.g., Refs. [5,6,7,8]).

In [5] and [6], the results of [4] are extended to multivariate spherically symmetric channels, in *n* dimensions and 2 dimensions, respectively. In both papers, the inputs are subject to average and peak radial constraints. It is shown that it is optimal to concentrate the input on a finite number of concentric shells.

In [7] and [8], the number and positioning of optimal concentric shells under a peak radial constraint is studied. In [7], the least restrictive amplitude constraint for which the optimal distribution is concentrated on a single sphere is found. In [8], it is shown that the number of shells grows at most quadratically in the amplitude constraint. A similar result is found for n=1 under an additional average power constraint.

While much of the prior work has focused on spherically symmetric channels, some research has considered spherically asymmetric channels. The case of inputs constrained to arbitrary compact sets and subject to a finite number of quadratic cost constraints as well as non-degenerate multivariate Gaussian noise is considered in [12]. It is concluded that the support of the capacity-achieving distribution must be “sparse”—that is, there must exist a not identically zero analytic function that is 0 on the support of the capacity-achieving distribution. Assuming otherwise leads to a contradiction by the *n*-dimensional Identity Theorem and Fourier analysis. These results, while quite general, do not consider either inputs of unbounded support or inputs subject to higher-moment constraints. Furthermore, outside of the special cases of n=1 and spherically symmetric channels, they do not explore a characterization of sparse sets in $R^{n}$.

In [13], MIMO channels with inputs that are restricted to compact sets, yet have no average power constraints, are considered. Using the Real-Analytic Identity Theorem and steps similar to [4], it is determined that the support of the optimal input distribution is nowhere dense in $R^{n}$ and has Lebesgue measure 0. For the case considered in [12] that coincides with this setup, [13] gives an instance of sparsity in terms of subsets of $R^{n}$, rather than analytic functions.

There has also been work dedicated to generalizing the classic quadratic average cost constraint. In [14], a scalar channel with the input subject to a combination of even-moment constraints and restrictions to compact or non-negative subsets of R is studied. It is shown that, in most of the cases considered, the support has a finite number of points.

In [15], a complex-valued non-dispersive optical channel is considered, where the input is subject to an average cost that grows super-quadratically in radius, a peak constraint, or both. The noise is taken to be circularly symmetric and, under these conditions, so is the optimal input. The number of concentric circles composing the support of the distribution is shown to be finite.

In this paper, we study an *n*-dimensional channel subject to non-degenerate Gaussian noise. The input can either take any value in $R^{n}$ or is restricted to a compact subset of $R^{n}$, and its norm is subject to even-moment constraints. The noise need not be spherically symmetric.

This paper gives a characterization of the capacity-achieving distribution to spherically asymmetric channels under peak and average power constraints that improves on prior work in three respects. Firstly, when our cases overlap with [12], our characterization of the capacity-achieving distribution is more detailed than the notion of sparsity used there. Secondly, our results apply to multivariate channels with inputs subject to even-moment constraints greater than 2. Thirdly, we consider both inputs that are restricted to compact sets and those that are allowed to take any value in $R^{n}$.

## 3. Results

In this section, we consider $R^{n}$-valued inputs subject to additive non-degenerate multivariate Gaussian noise. In Section 3.1, the capacity-achieving distribution, $F^{*}$, is formulated as the objective of an optimization problem; its support is then framed in terms of the zero set of a certain real-analytic function, which is dependent on $F^{*}$ and referred to as $s(\cdot ;F^{*})$. Section 3.2 finds an equivalent expression for $s(\cdot ;F^{*})$, which is an intermediary step to showing in Section 3.3 that $s(\cdot ;F^{*})$ is non-constant. Section 3.4 uses the result that $s(\cdot ;F^{*})$ is non-constant to show that the support of the capacity-achieving distribution is contained in a countable union of submanifolds of dimensions in the range {0,…,n−1}. This union is finite when the input is constrained to a compact subset of $R^{n}$. It is then shown that the support of the capacity-achieving input has Lebesgue measure 0 and is nowhere dense in $R^{n}$.

Appendix A is dedicated to showing the convexity and compactness of the optimization space used in Section 3.1. Appendix B establishes a pointwise characterization of $supp(F^{*})$, which justifies the definition of $s(\cdot ,F^{*})$. Appendix C provides integrability results, which are used throughout the paper. Appendix D shows that the objective functional is weak continuous, strictly concave, and weak differentiable on the optimization space. Appendix E shows that $s(\cdot ,F^{*})$ has an analytic extension to $C^{n}$. Finally, Appendix F supports Section 3.3 by finding bounds for certain functions.

As a first step towards defining the set of feasible input distributions, let $F(R^{n})$ be the set of finite Borel measures on $R^{n}$. Note that $F(R^{n})$ is contained in the set of finite signed Borel measures on $R^{n}$, which has an intrinsic vector space structure and can be equipped with a norm [16]. Since $F(R^{n})$ lies within a normed vector space, the convexity and compactness of its subsets can be discussed.

The possibility that the transmitter is unable to produce arbitrary signals in $R^{n}$ is modeled by restricting the input to an alphabet $A\subseteq R^{n}$. Denote the set of distributions for which the associated random variable is almost surely in A by

(1) $$F_{n}\left(A\right)≜\left\{F\in F\left(R^{n}\right)|F\left(A\right)=1\right\}.$$

Two cases for A are considered:

1. $A=R^{n}$;
2. $A⊊R^{n}$ is compact.

In addition to the restriction to A, a radial even-moment constraint is associated with the input. For $k\in Z_{>0}$, the input must belong to the set

(2) $$P_{n}\left(A,k,a\right)≜\{F\in F_{n}\left(A\right)|E_{X∼F}{[∥X∥}^{2k}]\;\leq \;a\}.$$

The resulting channel model, with input $X∼F\in P_{n}\left(A,k,a\right)$, is

(3) Y=AX+N,

where Y and N∼N(0,Σ) are output and noise, respectively, and A is an invertible matrix known to the transmitter and receiver. It is assumed that the noise covariance matrix Σ is positive-definite.

We will simplify the analysis of (3) by showing that no generality is lost in assuming that $A=I_{n}$, the *n*-dimensional identity matrix, and Σ is diagonal. Since Σ is positive-definite and A is invertible, the positive-definite matrix $A^{-1}\Sigma {\left(A^{-1}\right)}^{T}$ can be diagonalized by the orthogonal matrix Q.

Now, multiplying the output Y in (3) by $QA^{-1}$, the receiver obtains

(4) $$\begin{array}{cc} \tilde{Y} & ≜QA^{-1}Y \end{array}$$

(5) $$\begin{array}{cc} \; & =\tilde{X}+\tilde{N}, \end{array}$$

where $\tilde{X}=QX$ and the covariance matrix of $\tilde{N}=QA^{-1}N$ is diagonal. Since $QA^{-1}$ is invertible, $I\left(X;Y\right)=I\left(\tilde{X};\tilde{Y}\right)$. Furthermore, since Q is orthogonal,

(6) $$E[∥\tilde{X}{∥}^{2k}]{=E[∥X∥}^{2k}]$$

and the set {Qx|x∈A} is merely a rotated version of A. Hence, no generality is lost by dropping the “∼” and adopting the following channel model for the remainder of the paper:

(7) Y=X+N,

where N∼N(0,Σ) and Σ is diagonal with entries $0<\sigma_{1}^{2}\leq \sigma_{2}^{2}\leq \ldots \leq \sigma_{n}^{2}$. The density of N is denoted by $p_{N}\left(\cdot \right)$.

### 3.1. Optimization Problem

By Theorem 3.6.2 of [17], the capacity of the channel in (7) is given by the optimization problem

(8) $$C=\underset{X∼F\in P_{n}\left(A,k,a\right)}{\sup}I\left(X;Y\right)=\underset{E_{X∼F}{[∥X∥}^{2k}]\leq a}{\underset{F\in F_{n}\left(A\right)}{\sup}}I\left(X;Y\right).$$

Since the relationship between Y, X, and N is known, the mutual information is a function of the distribution of X alone. Thus, the mutual information induced between X∼F and Y will be denoted by I(F). Similarly, we express the even-moment constraint in terms of a functional $g_{k}:F_{n}\left(A\right)\to R\cup \left\{\infty \right\}$ given by

(9) $$g_{k}\left(F\right)≜\int_{A}{∥x∥}^{2k}\;dF(x)-a,$$

where $g_{k}\left(F\right)\leq 0$ is equivalent to $E_{X∼F}{[∥X∥}^{2k}]\;\leq \;a$. Rewriting (8) in terms of I(·) and $g_{k}\left(\cdot \right)$ yields

(10) $$C=\underset{F\in P_{n}\left(A,k,a\right)}{\sup}I\left(F\right)=\underset{g_{k}\left(F\right)\leq 0}{\underset{F\in F_{n}\left(A\right)}{\sup}}I\left(F\right).$$

Much of the appendix is dedicated to understanding properties of the problem presented in (10). It is shown in Theorem A1 that $P_{n}\left(A,k,a\right)$ is convex and compact. Furthermore, by Theorems A3 and A4, I(·) is a weakly continuous and strictly concave function on $P_{n}\left(A,k,a\right)$. Therefore, the supremum is achieved by a unique input distribution $F^{*}\in P_{n}\left(A,k,a\right)$ (see, e.g., Appendix C of [14])—that is,

(11) $$C=\max_{F\in P_{n}\left(A,k,a\right)}I\left(F\right)=\underset{g_{k}\left(F\right)\leq 0}{\max_{F\in F_{n}\left(A\right)}}I\left(F\right)=I\left(F^{*}\right).$$

We use the notation $X^{*}$ to describe a capacity-achieving input directly (i.e., $X^{*}∼F^{*}$).

Before proceeding, we require some definitions and notations. In the first definition, and throughout the paper, for $x\in R^{n}$ and r>0, we denote the ball of radius *r* centered at x by $B_{r}\left(x\right)\subseteq R^{n}$. We will denote the closure of $B_{r}\left(x\right)$ by $\bar{B_{r}}\left(x\right)$. The output density induced by an input X∼F is denoted p(·;F).

**Definition 1.**  *Let V be a random variable with alphabet $A\subseteq R^{n}$. Then, the support of V is the set given by*

(12) $$supp\left(V\right)≜\{x\in A|\forall r>0,P\left\{V\in B_{r}\left(x\right)\right\}>0\}.$$

*If V has distribution $F_{V}$, we may alternatively refer to $supp\left(F_{V}\right)≜supp\left(V\right)$.*

**Definition 2.**  *For $F\in F_{n}\left(A\right)$, the output differential entropy is given by*

(13) $$h_{Y}\left(F\right)≜-\int_{R^{n}}p\left(y;F\right)\ln p(y;F)\;dy$$

*and the marginal entropy density at $x\in R^{n}$ is given by*

(14) $$h\left(x;F\right)≜-\int_{R^{n}}p_{N}\left(y-x\right)\ln p(y;F)\;dy,$$

*whenever the integrals exist.*

The relationship between the differential entropy and marginal entropy density can be seen as follows. For any b>0 and $F_{1},F_{2}\in P_{n}\left(A,k,b\right)$, we have that

(15) $$\begin{array}{cc} \int_{A}h\left(x;F_{1}\right)\;dF_{2}\left(x\right) & =-\int_{A}\int_{R^{n}}p_{N}\left(y-x\right)\ln p(y;F_{1})\;dy\;dF_{2}\left(x\right) \end{array}$$

(16) $$\begin{array}{cc} \; & =-\int_{R^{n}}\int_{A}p_{N}\left(y-x\right)\;dF_{2}\left(x\right)\ln p(y;F_{1})\;dy \end{array}$$

(17) $$\begin{array}{cc} \; & =-\int_{R^{n}}p\left(y;F_{2}\right)\ln p(y;F_{1})\;dy. \end{array}$$

The equivalence of (15)–(17) is due to Fubini–Tonelli [18] and Lemma A4. If $F=F_{1}=F_{2}$, then

(18) $$\int_{A}h\left(x;F\right)\;dF(x)=h_{Y}\left(F\right).$$

Lastly, define

(19) $$\begin{array}{cc} Q_{n}\left(A,k,a\right) & ≜\underset{b\geq a}{⋃}P_{n}\left(A,k,b\right) \end{array}$$

and let $s\left(\cdot ;F^{*}\right):R^{n}\to R$ be given by

(20) $$\begin{array}{cc} s(x;F^{*}) & ≜{\gamma (∥x∥}^{2k}-a)+C+h\left(N\right)-h\left(x;F^{*}\right) \end{array}$$

(21) $$\begin{array}{cc} \; & ={\gamma (∥x∥}^{2k}-a)+C+h\left(N\right)+\int_{R^{n}}p_{N}\left(y-x\right)\ln p(y;F^{*})\;dy. \end{array}$$

Since $F^{*}\in P_{n}\left(A,k,a\right)$, we have that $h(x;F^{*})$ is finite for all $x\in R^{n}$ and conclude that $s(x;F^{*})$ is also finite for all $x\in R^{n}$. Furthermore, by Lemma A8, $s(\cdot ;F^{*})$ can be extended to a complex analytic function for all $z\in C^{n}$; hence, it is continuous.

The remainder of Section 3.1 consists of two steps:

1. We show that $F^{*}$ solves (11) if and only if there exists γ≥0 such that
  (22) $$\int_{R^{n}}h\left(x;F^{*}\right)\;dF\left(x\right)-C-h\left(N\right)-\gamma g_{k}\left(F\right)\leq 0,$$
  for all $F\in Q_{n}\left(A,k,a\right)$.
2. We show that, for the choice of γ in (22), the inequality in (22) is equivalent to the condition that for all x∈A,
  (23) $$h\left(x;F^{*}\right)\leq {\gamma (∥x∥}^{2k}-a)+C+h\left(N\right),$$
  and if $x\in supp(F^{*})$, then
  (24) $$h\left(x;F^{*}\right)={\gamma (∥x∥}^{2k}-a)+C+h\left(N\right).$$
  For x∈A, (24) is satisfied if and only if $s(x;F^{*})=0$.

To establish (22), we will first use a Lagrange multiplier to reformulate (11) as an unconstrained problem over $Q_{n}\left(A,k,a\right)$. We will then obtain (22) by taking the weak derivative of the resulting objective functional and applying a Karush–Kuhn–Tucker condition for optimality. We choose to work in the space $Q_{n}\left(A,k,a\right)$ since, when $A=R^{n}$, the functionals I(·) and $g_{k}\left(\cdot \right)$ are not weakly differentiable on the larger space $F_{n}\left(A\right)=F_{n}\left(R^{n}\right)$.

Since $\left\{F\in Q_{n}\left(A,k,a\right)|g_{k}\left(F\right)\leq 0\right\}=P_{n}\left(A,k,a\right)$, (11) can equivalently be written as

(25) $$C=\underset{g_{k}\left(F\right)\leq 0}{\max_{F\in Q_{n}\left(A,k,a\right)}}I\left(F\right)=I\left(F^{*}\right),$$

where $F^{*}$ is the same as in (11). By Theorem A5, $g_{k}\left(\cdot \right)$ is convex. Moreover, letting $F_{s}\in Q_{n}\left(A,k,a\right)$ be a Heaviside step function at $0\in R^{n}$, $F_{s}$ is an interior point of the feasible region since $g_{k}\left(F_{s}\right)=-a<0$. Since $Q_{n}\left(A,k,a\right)$ is convex by Lemma A1, there exists γ≥0 such that

(26) $$C=\max_{F\in Q_{n}\left(A,k,a\right)}J_{\gamma }\left(F\right)=J_{\gamma }\left(F^{*}\right),$$

where

(27) $$J_{\gamma }\left(F\right)≜I\left(F\right)-\gamma g_{k}\left(F\right),$$

and $\gamma g_{k}\left(F^{*}\right)=0$ (see e.g., Appendix C of [14]). Furthermore, for an arbitrary b≥a, $F^{*}\in P_{n}\left(A,k,b\right)\subseteq Q_{n}\left(A,k,a\right)$. Therefore, for this choice of γ, we also have

(28) $$C=\max_{F\in P_{n}\left(A,k,b\right)}J_{\gamma }\left(F\right)=J_{\gamma }\left(F^{*}\right).$$

By Theorems A5 and A6, $J_{\gamma }\left(\cdot \right)$ has a weak derivative at $F^{*}$ in the direction of any $F\in P_{n}\left(A,k,b\right)$ given by

(29) $$\begin{array}{cc} J_{\gamma }^{\prime }\left(F^{*},F\right) & =I^{\prime }\left(F^{*},F\right)-\gamma g_{k}^{\prime }\left(F^{*},F\right) \end{array}$$

(30) $$\begin{array}{cc} \; & =\int_{A}h\left(x;F^{*}\right)\;dF(x)-h_{Y}\left(F^{*}\right)-\gamma \left(g_{k}\left(F\right)-g_{k}\left(F^{*}\right)\right) \end{array}$$

(31) $$\begin{array}{cc} \; & =\int_{A}h\left(x;F^{*}\right)\;dF(x)-h_{Y}\left(F^{*}\right)-\gamma g_{k}\left(F\right), \end{array}$$

where (31) is due to $\gamma g_{k}\left(F^{*}\right)=0$. Substituting $C=h_{Y}\left(F^{*}\right)-h\left(N\right)$ gives

(32) $$J_{\gamma }^{\prime }\left(F^{*},F\right)=\int_{A}h\left(x;F^{*}\right)\;dF(x)-C-h\left(N\right)-\gamma g_{k}\left(F\right),$$

where the differential entropy of the noise $h\left(N\right)=\frac{1}{2}\ln\left|2\pi e\Sigma \right|$ is finite since Σ is positive definite.

Now, $J_{\gamma }\left(\cdot \right)$ is the difference between a strictly concave function (see Theorem A4) and a convex function (due to Theorem A5 and the non-negativity of γ). Therefore, $J_{\gamma }\left(\cdot \right)$ is strictly concave and $F^{*}$ is optimal if and only if, for all $F\in P_{n}\left(A,k,b\right)$,

(33) $$J_{\gamma }^{\prime }\left(F^{*},F\right)=\int_{A}h\left(x;F^{*}\right)\;dF(x)-C-h\left(N\right)-\gamma g_{k}\left(F\right)\leq 0.$$

However, b≥a is arbitrary and each $F\in Q_{n}\left(A,k,a\right)$ satisfies $F\in P_{n}\left(A,k,b\right)$ for some b≥a. Therefore, $F^{*}$ is optimal if and only if, for all $F\in Q_{n}\left(A,k,a\right)$,

(34) $$J_{\gamma }^{\prime }\left(F^{*},F\right)=\int_{A}h\left(x;F^{*}\right)\;dF(x)-C-h\left(N\right)-\gamma g_{k}\left(F\right)\leq 0,$$

which is the statement we sought to show in (22).

The condition (34) is on the capacity-achieving distribution $F^{*}$ itself. Since our objective is to characterize $supp(F^{*})$, we find an equivalent condition to (34) to describe the behavior of $F^{*}$ at individual points in the input alphabet A. Thus, by (34) and Theorem A2, for all $x\in A\subseteq R^{n}$,

(35) $$s\left(x;F^{*}\right)={\gamma (∥x∥}^{2k}-a)+C+h\left(N\right)+\int_{R^{n}}p_{N}\left(y-x\right)\ln p(y;F^{*})\;dy\geq 0,$$

and if $x\in supp(F^{*})$, then

(36) $$\begin{array}{c} s\left(x;F^{*}\right)={\gamma (∥x∥}^{2k}-a)+C+h\left(N\right)+\int_{R^{n}}p_{N}\left(y-x\right)\ln p(y;F^{*})\;dy=0. \end{array}$$

The rest of Section 3 is dedicated to exploiting the relationship

(37) $$supp\left(F^{*}\right)\subseteq Z\left(s;F^{*}\right)\cap A,$$

where $Z(s;F^{*})$ is the zero set of $s(\cdot ,F^{*})$.

### 3.2. Hilbert Space and Hermite Polynomial Representation

In this subsection, an equivalent expression for (36) is found by viewing the integral as an inner product in a Hilbert space and writing $\ln p(\cdot ;F^{*})$ in terms of a Hermite polynomial basis for that space. Hermite polynomial bases are well-suited to analysis of Gaussian noise channels and they have been used in a number of information-theoretic papers (see, e.g., Refs. [14,19]).

Consider the Hilbert space

(38) $$L_{p_{N}}^{2}\left(R^{n}\right)≜\{\xi :R^{n}\to R|\int_{R^{n}}\xi^{2}\left(x\right)p_{N}\left(x\right)\;dx<\infty \},$$

equipped with inner product

(39) $${\langle \xi ,\psi \rangle }_{L_{p_{N}}^{2}\left(R^{n}\right)}≜\int_{R^{n}}\xi \left(x\right)\psi \left(x\right)p_{N}\left(x\right)\;dx.$$

The inner product’s subscript is omitted when the space can be inferred.

Since the components of N are independent, with $N_{i}$ having variance $\sigma_{i}^{2}>0$, the density of N factors into

(40) $$\begin{array}{cc} p_{N}\left(y\right) & =\prod_{i=1}^{n}p_{N_{i}}\left(y_{i}\right) \end{array}$$

(41) $$\begin{array}{cc} \; & =\prod_{i=1}^{n}\frac{1}{\sqrt{2\pi \sigma_{i}^{2}}}e^{-\frac{1}{2\sigma_{i}^{2}}y_{i}^{2}}. \end{array}$$

We will construct an orthogonal basis for $L_{p_{N}}^{2}\left(R^{n}\right)$ from orthogonal bases for the spaces $L_{p_{N_{i}}}^{2}\left(R\right)$.

First, with Z∼N(0,1), an orthogonal basis for $L_{p_{Z}}^{2}\left(R\right)$ is given by the Hermite polynomials ${\left\{H_{m}\right\}}_{m=0}^{\infty }$ [20], which are defined through the generating function

(42) $$e^{-\frac{1}{2}x^{2}+yx}=\sum_{m=0}^{\infty }H_{m}\left(y\right)\frac{x^{m}}{m!}.$$

For any $m\in Z_{\geq 0}$, the *m*th Hermite polynomial has degree *m* and a positive leading coefficient. Next, for each i∈{1,…,n} and $m_{i}\in Z_{\geq 0}$, define the stretched Hermite polynomials

(43) $$H_{m_{i}}\left(y;\sigma_{i}^{2}\right)≜\frac{1}{\sigma_{i}^{m_{i}}}H_{m}\left(\frac{y}{\sqrt{\sigma_{i}^{2}}}\right).$$

The inner product of $L_{p_{N_{i}}}^{2}\left(R\right)$ is related to that of $L_{p_{Z}}^{2}\left(R\right)$ by

(44) $$\begin{array}{cc} {\langle \xi ,\psi \rangle }_{L_{p_{N_{i}}}^{2}\left(R\right)} & =\int_{-\infty }^{\infty }\frac{1}{\sqrt{2\pi \sigma_{i}^{2}}}e^{-\frac{1}{2\sigma_{i}^{2}}y^{2}}\xi \left(y\right)\psi \left(y\right)\;dy \end{array}$$

(45) $$\begin{array}{cc} \; & =\int_{-\infty }^{\infty }\frac{1}{\sqrt{2\pi }}e^{-\frac{1}{2}u^{2}}\xi \left(\sqrt{\sigma_{i}^{2}}u\right)\psi \left(\sqrt{\sigma_{i}^{2}}u\right)\;du \end{array}$$

(46) $$\begin{array}{cc} \; & ={\langle \xi_{\sqrt{\sigma_{i}^{2}}},\psi_{\sqrt{\sigma_{i}^{2}}}\rangle }_{L_{p_{Z}}^{2}\left(R\right)}, \end{array}$$

where $\xi_{\sqrt{\sigma_{i}^{2}}}$ and $\psi_{\sqrt{\sigma_{i}^{2}}}$ are versions of ξ and ψ that have been stretched horizontally by a factor of $1/\sqrt{\sigma_{i}^{2}}$. Substituting any $H_{m_{i}}\left(\cdot ,\sigma_{i}^{2}\right)$ and $H_{l_{i}}\left(\cdot ,\sigma_{i}^{2}\right)$ into (44) shows that the set ${\left\{H_{m_{i}}\left(\cdot ,\sigma_{i}^{2}\right)\right\}}_{m_{i}=0}^{\infty }$ is orthogonal. Furthermore, if there was a non-zero function (in an $L_{p_{N_{i}}}^{2}\left(R\right)$ sense) that had a zero $L_{p_{N_{i}}}^{2}\left(R\right)$ inner product with $H_{m_{i}}\left(\cdot ,\sigma_{i}^{2}\right)$ for each $m_{i}$, then a stretched version of this function would also have a zero $L_{p_{Z}}^{2}\left(R\right)$ inner product with $H_{m}$ for each *m*. This would contradict the completeness of the Hermite polynomials in $L_{p_{Z}}^{2}\left(R\right)$; hence, ${\left\{H_{m_{i}}\left(\cdot ,\sigma_{i}^{2}\right)\right\}}_{m_{i}=0}^{\infty }$ forms a basis for $L_{p_{N_{i}}}^{2}\left(R\right)$. Lastly, the stretched Hermite polynomials ${\left\{H_{m_{i}}\left(\cdot ,\sigma_{i}^{2}\right)\right\}}_{m_{i}=0}^{\infty }$ have the generating function

(47) $$e^{-\frac{1}{2\sigma_{i}^{2}}x^{2}+\frac{1}{\sigma_{i}^{2}}yx}=\sum_{m_{i}=0}^{\infty }H_{m_{i}}\left(y;\sigma_{i}^{2}\right)\frac{x^{m_{i}}}{m_{i}!}.$$

Now, $L_{p_{N}}^{2}\left(R^{n}\right)$ is isomorphic to the tensor product of the $L_{p_{N_{i}}}^{2}\left(R\right)$ spaces, i∈{1,…,n}; consequently, $\left\{H_{m}\left(\cdot ;\sigma^{2}\right)|m\in Z_{\geq 0}^{n}\right\}$ forms an orthonormal basis for $L_{p_{N}}^{2}\left(R^{n}\right)$ [21], where

(48) $$H_{m}\left(y;\sigma^{2}\right)≜\prod_{i=1}^{n}H_{m_{i}}\left(y_{i};\sigma_{i}^{2}\right).$$

Since, by Lemma A2, $\ln p(\cdot ;F^{*})\in L_{p_{N}}^{2}\left(R^{n}\right)$, there exist constants ${\left\{c_{i}^{\prime }\right\}}_{i\in Z_{\geq 0}^{n}}$ for which

(49) $$\ln p(y;F^{*})=\sum_{i\in Z_{\geq 0}^{n}}c_{i}^{\prime }H_{i}\left(y;\sigma^{2}\right),$$

where equality is in an $L_{p_{N}}^{2}\left(R^{n}\right)$ sense. Then, substituting (47), and using the notations

(50) $$m!≜m_{1}!\cdots m_{n}!,$$

and

(51) $$x^{m}≜x_{1}^{m_{1}}\cdots x_{n}^{m_{n}}$$

for $m\in Z_{\geq 0}^{n}$ and $x\in R^{n}$, we write

(52) $$\begin{array}{cc} e^{-\frac{1}{2}x^{T}\Sigma^{-1}x+x^{T}\Sigma^{-1}y} & =\prod_{i=1}^{n}e^{-\frac{1}{2\sigma_{i}^{2}}x_{i}^{2}+\frac{1}{\sigma_{i}^{2}}y_{i}x_{i}} \end{array}$$

(53) $$\begin{array}{cc} \; & =\prod_{i=1}^{n}\sum_{m_{i}=0}^{\infty }H_{m_{i}}\left(y_{i};\sigma_{i}^{2}\right)\frac{x_{i}^{m_{i}}}{m_{i}!} \end{array}$$

(54) $$\begin{array}{cc} \; & =\sum_{m\in Z_{\geq 0}^{n}}\prod_{i=1}^{n}H_{m_{i}}\left(y_{i};\sigma_{i}^{2}\right)\frac{x_{i}^{m_{i}}}{m_{i}!} \end{array}$$

(55) $$\begin{array}{cc} \; & =\sum_{m\in Z_{\geq 0}^{n}}H_{m}\left(y;\sigma^{2}\right)\frac{x^{m}}{m!}. \end{array}$$

Substituting (49) and (55) into the integral term in (36) yields

(56) $$\begin{array}{cc} \int_{R^{n}}p_{N}\left(y-x\right)\ln p(y;F^{*})\;dy\; & =\int_{R^{n}}p_{N}\left(y\right)e^{-\frac{1}{2}x^{T}\Sigma^{-1}x+x^{T}\Sigma^{-1}y}\sum_{i\in Z_{\geq 0}^{n}}c_{i}^{\prime }H_{i}\left(y;\sigma^{2}\right)\;dy \end{array}$$

(57) $$\begin{array}{cc} \; & =\int_{R^{n}}p_{N}\left(y\right)\sum_{m\in Z_{\geq 0}^{n}}\frac{x^{m}}{m!}H_{m}\left(y;\sigma^{2}\right)\sum_{i\in Z_{\geq 0}^{n}}c_{i}^{\prime }H_{i}\left(y;\sigma^{2}\right)\;dy \end{array}$$

(58) $$\begin{array}{cc} \; & =\sum_{m\in Z_{\geq 0}^{n}}\frac{x^{m}}{m!}\sum_{i\in Z_{\geq 0}^{n}}c_{i}^{\prime }\langle H_{m}\left(y;\sigma^{2}\right),H_{i}\left(y;\sigma^{2}\right)\rangle \end{array}$$

(59) $$\begin{array}{cc} \; & =\sum_{m\in Z_{\geq 0}^{n}}c_{m}x^{m}, \end{array}$$

where $c_{m}=c_{m}^{\prime }/\rho_{m}$, with

(60) $$\infty >\rho_{m}≜\frac{m!}{\langle H_{m}\left(y;\sigma^{2}\right),H_{m}\left(y;\sigma^{2}\right)\rangle }>0.$$

This simplification to a polynomial will be helpful since the cost function associated with the even-moment constraint is also a polynomial. This relationship is exploited in Section 3.3.

### 3.3. Non-Constancy of $s(\cdot ;F^{*})$

Recall the relationship $supp\left(F^{*}\right)\subseteq Z\left(s;F^{*}\right)\cap A$ from (37). Since $supp(F^{*})\neq \emptyset$, $s(\cdot ;F^{*})$ has at least one zero, it is constant if and only if $Z\left(s;F^{*}\right)=R^{n}.$ This subsection is dedicated to showing the latter equivalent condition. The immediate implication is that $supp(F^{*})$ is a strict subset of $R^{n}$; however, the fact that $s(\cdot ;F^{*})$ is a non-zero real-analytic function will be used in Section 3.4 to prove the main results.

By way of contradiction, suppose that, for all $x\in R^{n}$, $s(x;F^{*})=0$. Substituting (59) into (36), this is equivalent to

(61) $$\begin{array}{cc} \left[\gamma a-C-h\left(N\right)\right]-\gamma (\sum_{i=1}^{n}x_{i}^{2}{)}^{k} & =\sum_{m\in Z_{\geq 0}^{n}}c_{m}x_{1}^{m_{1}}\ldots x_{n}^{m_{n}}, \end{array}$$

for all $x\in R^{n}$. The discussion proceeds in two cases: k=1 and k>1.

Case k=1:

In the case that k=1 and $A=R^{n}$, $X^{*}$ is known to be Gaussian [22] and there is no contradiction with (61). Therefore, for k=1, we focus only on compact input alphabets $A⊊R^{n}$.

With k=1 and $A⊊R^{n}$, ref. (61) reduces to

(62) $$\begin{array}{cc} \left[\gamma a-C-h\left(N\right)\right]-\gamma \sum_{i=1}^{n}x_{i}^{2} & =\sum_{m\in Z_{\geq 0}^{n}}c_{m}x_{1}^{m_{1}}\ldots x_{n}^{m_{n}}, \end{array}$$

for all $x\in R^{n}$. Let $e_{i}$ be the *i*′th row of the n×n identity matrix and let $0\in Z_{\geq 0}^{n}$ be the all zero vector. Since (62) holds for all $x\in R^{n}$, matching coefficients gives

(63) $$c_{m}=\left\{\begin{array}{cc} \gamma a-C-h(N), & \mathrm{if}\;m=0\\ -\gamma , & \mathrm{if}\;m=2e_{i},\;i\in \left\{1,\ldots ,n\right\}\\ 0, & \mathrm{otherwise}. \end{array}\right.$$

Since, for each i∈{1,…,n} and $m\in Z_{\geq 0}^{n}$, $H_{m_{i}}\left(y_{i};\sigma_{i}^{2}\right)$ has degree $m_{i}$ and a positive leading coefficient,

(64) $$H_{m}\left(y;\sigma^{2}\right)=\prod_{i=1}^{n}H_{m_{i}}\left(y_{i};\sigma_{i}^{2}\right)$$

also has degree $m_{i}$ in $y_{i}$ and the unique term with total degree $d\left(m\right)=m_{1}+\ldots +m_{n}$ is $y^{m_{1}}\cdots y^{m_{n}}$, which has a positive coefficient. Therefore, the polynomials present in the sum are of the form

(65) $$H_{0}\left(y;\sigma^{2}\right)=\kappa_{0}>0$$

and, for i∈{1,…,n},

(66) $$H_{2e_{i}}\left(y;\sigma^{2}\right)=\kappa_{2e_{i}}y_{i}^{2}+\alpha_{2e_{i}}y_{i}+\beta_{2e_{i}}.$$

The constants $\kappa_{0}$ and $\kappa_{2e_{i}}$ are positive, while $\alpha_{2e_{i}}$ and $\beta_{2e_{i}}$ are real. Substituting this and the identity $c_{m}^{\prime }=c_{m}\rho_{m}$ into (49) yields

(67) $$\begin{array}{cc} \ln p(y;F^{*}) & =c_{0}\rho_{0}H_{0}\left(y;\sigma^{2}\right)+\sum_{i=1}^{n}c_{2e_{i}}\rho_{2e_{i}}H_{2e_{i}}\left(y;\sigma^{2}\right) \end{array}$$

(68) $$\begin{array}{cc} \; & =\kappa_{0}\rho_{0}\left[\gamma a-C-h\left(N\right)\right]-\gamma \sum_{i=1}^{n}\rho_{2e_{i}}\left(\kappa_{2e_{i}}y_{i}^{2}+\alpha_{2e_{i}}y_{i}+\beta_{2e_{i}}\right), \end{array}$$

or equivalently,

(69) $$p\left(y;F^{*}\right)=e^{\kappa_{0}\rho_{0}\left[\gamma a-C-h\left(N\right)\right]}\prod_{i=1}^{n}e^{-\gamma \rho_{2e_{i}}\left(\kappa_{2e_{i}}y_{i}^{2}+\alpha_{2e_{i}}y_{i}+\beta_{2e_{i}}\right)}.$$

By definition, γ≥0; however, γ=0 results in a constant density on $R^{n}$, which is invalid. Then, it must be the case that γ>0. Thus, the output achieved by $X^{*}$, $Y^{*}≜X^{*}+N$ has independent Gaussian components. Since $X^{*}$ and N are independent and N is an *n*-variate Gaussian random variable, $X^{*}$ must either be an *n*-variate Gaussian random variable or be almost surely equal to some $x_{0}\in R^{n}$. In the former case, $X^{*}$ violates the stipulation that the input alphabet A is compact and contradicts the assumption that $s(\cdot ;F^{*})$ is identically 0. In the latter case, $supp\left(F^{*}\right)=\left\{x_{0}\right\}$ is trivial and satisfies the main results of the paper.

Case k>1:

For the case k>1, we derive a contradiction to (61) using results on the rate of decay of a function compared with that of its Fourier transform to conclude that $s(\cdot ;F^{*})$ is not identically 0.

**Lemma 1.**  *Let $U\in R^{n}$ have, for some β>0, a characteristic function satisfying*

(70) $$\left|\phi_{U}\left(\omega \right)\right|≜\left|E\left[e^{i\omega^{T}U}\right]\right|\leq e^{-\frac{{\beta ∥\omega ∥}^{2}}{2}}$$

*for all $\omega \in R^{n}$. Let V be a random variable independent of U. Then, the characteristic function of W=U+V satisfies, for all $\omega \in R^{n}$,*

(71) $$\left|\phi_{W}\left(\omega \right)\right|\leq e^{-\frac{{\beta ∥\omega ∥}^{2}}{2}}.$$

**Proof.**  By the independence of U and V, and the fact that characteristic functions have pointwise moduli upper-bounded by 1,

(72) $$\begin{array}{cc} |\phi_{W}\left(\omega \right)| & =\;|\phi_{U}\left(\omega \right)||\phi_{V}\left(\omega \right)| \end{array}$$

(73) $$\begin{array}{cc} \; & \leq |\phi_{V}\left(\omega \right)|e^{-\frac{{\beta ∥\omega ∥}^{2}}{2}} \end{array}$$

(74) $$\begin{array}{cc} \; & \leq e^{-\frac{{\beta ∥\omega ∥}^{2}}{2}}. \end{array}$$

□

**Lemma 2.**  *Let $U\in R^{n}$ have, for some constant β>0, a characteristic function satisfying*

(75) $$\left|\phi_{U}\left(\omega \right)\right|≜\left|E\left[e^{i\omega^{T}U}\right]\right|\leq e^{-\frac{{\beta ∥\omega ∥}^{2}}{2}}$$

*for all $\omega \in R^{n}$. Let V be a random variable independent of U and W=U+V have density $p_{W}\left(\cdot \right)$. If there exist positive constants α and K such that, for all $x\in R^{n}$,*

(76) $$p_{W}\left(x\right)\leq Ke^{-{\alpha ∥x∥}^{2}},$$

*then αβ≤0.5.*

**Proof.**  Apply Lemma 1 and Theorem 4 of [23], noting that an identically 0 function cannot be a density. □

We make use of Lemma 2 by setting U=N, $V=X^{*}$, and $W=Y^{*}$ and deriving a contradiction to the assumption that $s(\cdot ;F^{*})$ is identically 0. Note that, using Rayleigh quotients, the modulus of the characteristic function of N can be upper-bounded for any $\omega \in R^{n}$ by

(77) $$\left|E\left[e^{i\omega^{T}N}\right]\right|=e^{-\frac{1}{2}\omega^{T}\Sigma \omega }\leq e^{-\frac{1}{2}\sigma_{1}^{2}{∥\omega ∥}^{2}}.$$

That is, the characteristic function of N satisfies (75) with $\beta =\sigma_{1}^{2}$.

To complete the contradiction, we show that there exists α>0.5/β and $K_{\alpha }>0$ such that $p(\cdot ;F^{*})$ satisfies the bound in (76). The assumption that $s(\cdot ;F^{*})$ is identically 0 yields (61); substituting the Multinomial Theorem,

(78) $$\left[\gamma a-C-h\left(N\right)\right]-\gamma \sum_{k_{1}+\ldots +k_{n}=k}\frac{k!}{k_{1}!\cdots k_{n}!}x_{1}^{2k_{1}}\cdots x_{n}^{2k_{n}}=\sum_{m\in Z_{\geq 0}^{n}}c_{m}x_{1}^{m_{1}}\cdots x_{n}^{m_{n}}.$$

By coefficient matching in (78), the set of non-zero coefficients, other than $c_{0}$, is indexed by the set

(79) $$M≜\{m\in Z_{\geq 0}^{n}|\sum_{i=1}^{n}m_{i}=2k\;\mathrm{and}\;m_{i}\;\mathrm{is}\;\mathrm{even}\;\forall i\in \left\{1,\ldots ,n\right\}\}.$$

Furthermore, for m∈M,

(80) $$c_{m}=-\gamma \frac{(\sum_{i=1}^{n}m_{i}/2)!}{\left(m_{1}/2\right)!\cdots \left(m_{n}/2\right)!}$$

Therefore, substituting (80) into (49),

(81) $$\begin{array}{cc} p(y;F^{*}) & =e^{\kappa_{0}\rho_{0}\left(\gamma a-C-h\left(N\right)\right)}e^{\sum_{m\in M}c_{m}\rho_{m}H_{m}\left(y;\sigma^{2}\right)}. \end{array}$$

for some positive constant $\kappa_{0}=H_{0}\left(y;\sigma^{2}\right)$. As with the case k=1, γ=0 results in a constant output density over $R^{n}$ and can be disregarded as a possibility. Thus, for each m∈M, we have

(82) $$c_{m}<0.$$

With $W=Y^{*}$ and α>0, showing that there exists $K_{\alpha }$ for which (76) holds, is equivalent to showing that

(83) $$\begin{array}{c} \frac{p(y;F^{*})}{e^{-{\alpha ∥y∥}^{2}}}=e^{\kappa_{0}\rho_{0}\left(\gamma a-C-h\left(N\right)\right)}e^{\sum_{m\in M}c_{m}\rho_{m}H_{m}\left(y;\sigma^{2}\right)+\alpha \sum_{i=1}^{n}y_{i}^{2}} \end{array}$$

is bounded. This, in turn, is equivalent to showing that the polynomial in the exponent,

(84) $$q_{\alpha }\left(y\right)≜\sum_{m\in M}c_{m}\rho_{m}H_{m}\left(y;\sigma^{2}\right)+\alpha \sum_{i=1}^{n}y_{i}^{2},$$

is upper bounded. We proceed by considering the degrees of the terms of $q_{\alpha }\left(y\right)$ to determine the behavior of (84) as ∥y∥ increases.

For each $m\in Z_{\geq 0}^{n}$ and i∈{1,…,n}, $H_{m}\left(y;\sigma^{2}\right)$ has degree $m_{i}$ in $y_{i}$. Furthermore, $H_{m}\left(y;\sigma^{2}\right)$ has total degree

(85) $$d\left(m\right)≜\sum_{i=1}^{n}m_{i}$$

and the unique highest degree term, $y_{1}^{m_{1}}\cdots y_{n}^{m_{n}}$, has coefficient $\kappa_{m}>0$. Note that, since k>1, and by the definition of M, $q_{\alpha }\left(\cdot \right)$ has total degree 2k≥4. Hence, ref. (84) can be rewritten as $q_{\alpha }\left(y\right)=q_{\alpha }^{\left(0\right)}\left(y\right)+q_{\alpha }^{\left(1\right)}\left(y\right)+q_{\alpha }^{\left(2\right)}\left(y\right)$, where

(86) $$\begin{array}{cc} q_{\alpha }^{\left(0\right)}\left(y\right) & ≜\sum_{i=1}^{n}c_{2ke_{i}}\rho_{2ke_{i}}\kappa_{2ke_{i}}y_{i}^{2k}=-\gamma \sum_{i=1}^{n}\rho_{2ke_{i}}\kappa_{2ke_{i}}y_{i}^{2k}, \end{array}$$

(87) $$\begin{array}{cc} \;q_{\alpha }^{\left(1\right)}\left(y\right) & ≜\sum_{\begin{array}{c} m\in M\\ m\notin \{2ke_{i}|i\in \left\{1,\ldots ,n\right\}\} \end{array}}c_{m}\rho_{m}\kappa_{m}y^{m}, \end{array}$$

and $q_{\alpha }^{\left(2\right)}\left(y\right)$ is the sum of the remaining terms, each with a total degree of at most 2k−2.

Note the following:

- For each $y\in R^{n}$, $q_{\alpha }^{\left(0\right)}\left(y\right)\leq 0$ and, by Lemma A9, the minimal value of $|q_{\alpha }^{\left(0\right)}\left(y\right)|$—evaluated on a sphere ∥y∥=L of radius L≥0—is at least $\gamma \min_{i\in \{1,\ldots ,n\}}\left\{\rho_{2ke_{i}}\kappa_{2ke_{i}}\right\}L^{2k}/n^{k}$.
- For each $y\in R^{n}$, we have that $q_{\alpha }^{\left(1\right)}\left(y\right)\leq 0$. Indeed, for each m∈M, $c_{m}<0$ by (82), $\rho_{m}>0$, and $\kappa_{m}>0$; further, for each i∈{1,…,n}, $m_{i}$ is even.
- The maximum value of $|q_{\alpha }^{\left(2\right)}\left(y\right)|$, evaluated on a sphere ∥y∥=L of radius L≥1, is at most $AL^{2k-2}$ for some A>0—that is, each term of $q_{\alpha }^{\left(2\right)}\left(y\right)$ is either of the form $\alpha y_{i}^{2}$ or $c_{m}\rho_{m}\nu_{m,l}y^{l}$ for some $m\in M,l\in Z_{\geq 0}^{n}$, and $\nu_{m,l}\in R$, where d(l)≤2k−2. Lemma A10 shows that these are no more than $\alpha L^{2}$ or $c_{m}\rho_{m}\nu_{m,l}L^{d(l)}$.

We conclude that, since $q_{\alpha }^{\left(0\right)}(y)\leq 0$ and $q_{\alpha }^{\left(1\right)}(y)\leq 0$ for all $y\in R^{n}$,

(88) $$\begin{array}{cc} \lim_{∥y∥\to \infty }q_{\alpha }\left(y\right) & =\lim_{∥y∥\to \infty }(q_{\alpha }^{\left(0\right)}\left(y\right)+q_{\alpha }^{\left(2\right)}\left(y\right))+\lim_{∥y∥\to \infty }q_{\alpha }^{\left(1\right)}\left(y\right) \end{array}$$

(89) $$\begin{array}{cc} \; & \leq \lim_{∥y∥\to \infty }(q_{\alpha }^{\left(0\right)}\left(y\right)+q_{\alpha }^{\left(2\right)}\left(y\right)) \end{array}$$

(90) $$\begin{array}{cc} \; & =-\infty . \end{array}$$

Thus, since $q_{\alpha }(y)$ is a continuous function that satisfies (90), it is bounded from above. Let $M_{q,\alpha }=\sup_{y\in R^{n}}q_{\alpha }\left(y\right)$ and

(91) $$K_{\alpha }=e^{\kappa_{0}\rho_{0}\left(\gamma a-C-h\left(N\right)\right)}e^{M_{q,\alpha }}.$$

Then, for all $y\in R^{n}$,

(92) $$p\left(y;F^{*}\right)\leq K_{\alpha }e^{-{\alpha ∥y∥}^{2}}.$$

Recall that, with $\sigma_{1}^{2}>0$, the smallest eigenvalue of Σ, and $\beta =\sigma_{1}^{2}$, the characteristic function of N satisfies (75). Let $\alpha =1/\sigma_{1}^{2}$ and choose $K_{\alpha }$ according to (91). Then, $p(y;F^{*})$ satisfies (92), yet αβ=1>0.5. Hence, the bound on the characteristic function of N given by (77) and the bound on the density of $Y^{*}$ given in (92) contradict Lemma 2. Therefore, the coefficient matching equation (78) cannot hold for all $x\in R^{n}$ and we conclude that, for k>1, $s(\cdot ;F^{*})$ cannot be identically 0 on $R^{n}$.

We summarize the results of the two cases, k=1 and k>1, in a theorem.

**Theorem 1.**  *Suppose that either*

*1.*  *$A⊊R^{n}$ is compact, or*
*2.*  *$A=R^{n}$, with k≠1.*

Then, either $supp\left(F^{*}\right)=\left\{x_{0}\right\}$ for some $x_{0}\in R^{n}$ or

(93) $$Z\left(s;F^{*}\right)⊊R^{n}.$$

An immediate consequence of Theorem 1 is that $supp(F^{*})$ is a strict subset of $R^{n}$. Recall from Section 3.1 that $s(\cdot ;F^{*})$ has an analytic extension to $C^{n}$. Therefore, Theorem 1 shows that $supp(F^{*})$ is “sparse" in the sense used by [12]—that is, there exists a non-zero function with an analytic extension to $C^{n}$ that is zero on $supp(F^{*})$. However, the primary importance of Theorem 1 is as an intermediary result that is used in Section 3.4 to obtain a better understanding of the structure of $supp(F^{*})$.

### 3.4. Main Results

In this section, we use geometry to show that $supp(F^{*})$ is contained in a countable disjoint union of submanifolds of dimensions ranging 0,…,n−1. Furthermore, this union is finite when A is compact. We then show that $supp(F^{*})$ has Lebesgue measure 0 and is nowhere dense in $R^{n}$.

The discussions in this section consider subsets of a vector’s components; so, for $x\in R^{n}$ and i∈{1,…,n}, we introduce the notation

(94) $$x^{i}≜\left(x_{1},\ldots ,x_{i}\right)\in R^{i}.$$

Recall from (37) in Section 3.1 that

(95) $$\begin{array}{cc} supp(F^{*}) & \subseteq Z(s;F^{*})\cap A. \end{array}$$

Since, by Lemma A8, $s(\cdot ;F^{*})$ has an analytic extension to $C^{n}$, it is real-analytic, which motivates us to study the geometry of zero sets of real-analytic functions. We start by restating Theorem 6.3.3 of [24] to the level that is needed in this paper.

**Theorem 2**  (Structure Theorem). *Let $\psi \left(\cdot \right):R^{n}\to R$ be a real-analytic function, where $\psi (0,\ldots ,0,x_{n})$ is not identically 0 in $x_{n}$. After a rotation of the coordinates $x_{1},\ldots ,x_{n-1}$, there exist constants $\delta_{m}$, m∈{1,…,n} such that with*

(96) $$Q≜\{x\in R^{n}||x_{m}|<\delta_{m}\;\forall m\in \left\{1,\ldots ,n\right\}\},$$

*we have*

(97) $$\left\{x\in Q|\psi \left(x\right)=0\right\}=\overset{n-1}{\underset{i=0}{⋃}}V_{i},$$

*where $V_{0}$ is either empty or contains only the origin and $V_{i}$, i∈{1,…,n−1}, is a finite disjoint union of i-dimensional submanifolds—that is, for each i∈{1,…,n−1}, there exists $n_{i}$ for which*

(98) $$V_{i}=\overset{n_{i}}{\underset{j=0}{⋃}}\Gamma_{i}^{j},$$

*where each $\Gamma_{i}^{j}$ is an i-dimensional submanifold. Furthermore, letting*

(99) $$Q_{i}≜\{x^{i}\in R^{i}||x_{m}\left|<\delta_{m}\;\forall m\in \left\{1,\ldots ,i\right\}\right\},$$

*there exists an open set $\Omega_{i}^{j}\subseteq Q_{i}$ and real-analytic functions $\alpha_{i}^{j,m}\left(\cdot \right)$, m∈{i+1,…,n}, on $\Omega_{i}^{j}$ for which*

(100) $$\Gamma_{i}^{j}=\left\{\left(x^{i},\alpha_{i}^{j,i+1}\left(x^{i}\right),\ldots ,\alpha_{i}^{j,n}\left(x^{i}\right)\right)\in R^{n}|x^{i}\in \Omega_{i}^{j}\right\}.$$

We apply Theorem 2 to characterize the zero set of $s(\cdot ;F^{*})$ in the form of (97) and obtain the following result.

**Theorem 3.**  *Suppose that either*

*1.*  *$A⊊R^{n}$ is compact, or*
*2.*  *$A=R^{n}$, with k≠1.*

*Then,*

(101) $$supp\left(F^{*}\right)\subseteq Z\left(s;F^{*}\right)\cap A=A\cap (\overset{n-1}{\underset{i=0}{⋃}}T_{i}),$$

*where $T_{0}$ is a countable union of isolated points and $T_{i}$, i∈{1,…,n−1}, is a countable disjoint union of i-dimensional submanifolds. Furthermore, if A is compact, these unions are finite.*

**Proof.**  First, note that, by Theorem 1, either $supp\left(F^{*}\right)=\left\{x_{0}\right\}$ for some $x_{0}\in R^{n}$ or $s(\cdot ;F^{*})$ is not identically 0 on $R^{n}$. In the former case, the result is trivially true; so, assume that $s(\cdot ;F^{*})$ is not identically 0 on $R^{n}$. Therefore, for any $q\in Q^{n}$, we can translate $s(\cdot ;F^{*})$ by q and rotate its coordinate system to apply Theorem 2—that is, there exists a sufficiently small open set $Q^{q}$ around q such that

(102) $$Z\left(s;F^{*}\right)\cap Q^{q}=\overset{n-1}{\underset{i=0}{⋃}}V_{i}^{q},$$

where the $V_{i}$ values are as in Theorem 2.

Since $Q^{n}$ is dense in $R^{n}$,

(103) $$A\subseteq \underset{q\in Q^{n}}{⋃}Q^{q}$$

Furthermore, if A is compact, the open cover ${\left\{Q^{q}\right\}}_{q\in Q^{n}}$ has a finite subcover ${\left\{Q^{q_{j}}\right\}}_{j=1}^{m}$—that is,

(104) $$A\subseteq \overset{m}{\underset{j=1}{⋃}}Q^{q_{j}}.$$

Defining the index set

(105) $$M≜\left\{\begin{array}{cc} Q^{n}, & A=R^{n}\\ {\left\{q_{j}\right\}}_{j=1}^{m}, & A⊊R^{n}\mathrm{is}\;\mathrm{compact}, \end{array}\right.$$

we obtain

(106) $$\begin{array}{cc} Z(s;F^{*})\cap A & =A\cap (\underset{q\in M}{⋃}\left(Z\left(s;F^{*}\right)\cap Q^{q}\right)) \end{array}$$

(107) $$\begin{array}{cc} \; & =A\cap (\underset{q\in M}{⋃}\overset{n-1}{\underset{i=0}{⋃}}V_{i}^{q}) \end{array}$$

(108) $$\begin{array}{cc} \; & =A\cap (\overset{n-1}{\underset{i=0}{⋃}}\underset{q\in M}{⋃}V_{i}^{q}). \end{array}$$

Since, for each q∈M, $V_{0}^{q}$ is either empty or a single point,

(109) $$T_{0}≜\underset{q\in M}{⋃}V_{0}^{q}$$

is a countable set of points and is finite when A is compact. Furthermore, each $V_{i}^{q}$, where i∈{1,…,n−1}, is itself a finite union of *i*-dimensional submanifolds. Hence,

(110) $$T_{i}≜\underset{q\in M}{⋃}V_{i}^{q}$$

is a countable union of *i*-dimensional submanifolds. When A is compact, this union is also finite. □

Note that Theorem 3 agrees with the results of [5] when the cases overlap. Indeed, consider the case in which A is a ball centered at the origin, k=1, and the noise covariance matrix $\Sigma =tI_{n}$, where $I_{n}$ is the n×n identity matrix and t>0. Then, ref. [5] shows that the capacity-achieving distribution is supported on a finite number of concentric (n−1)-spheres. Each (n−1)-sphere is an n−1 dimensional submanifold.

In the next two theorems, we show that $supp(F^{*})$ has Lebesgue measure 0 and is nowhere dense in $R^{n}$.

**Theorem 4.**  *Suppose that either*

*1.*  *$A⊊R^{n}$ is compact, or*
*2.*  *$A=R^{n}$, with k≠1.*

*Let μ(·) denote the n-dimensional Lebesgue measure. Then,*

(111) $$\mu (supp\left(F^{*}\right))=0.$$

**Proof.**  By Theorem 3, we have

(112) $$\begin{array}{cc} supp(F^{*}) & \subseteq A\cap (\overset{n-1}{\underset{i=0}{⋃}}T_{i}) \end{array}$$

(113) $$\begin{array}{cc} \; & \subseteq \overset{n-1}{\underset{i=0}{⋃}}\underset{q\in M}{⋃}V_{i}^{q}, \end{array}$$

where M is countable. Note that for each q∈M, $V_{0}^{q}$ is either empty or a single point; so, $\mu (V_{0}^{q})=0$. Furthermore, for each q∈M and i∈{1,…,n−1}, $V_{i}^{q}$ is a finite disjoint union of $n_{i}^{q}$*i*-dimensional submanifolds, and for i≤n−1, each submanifold has Lebesgue measure 0. Therefore, $\mu (supp\left(F^{*}\right))=0$. □

We will now define the notion of a subset being nowhere dense in its superset and show that $supp(F^{*})$ is nowhere dense in $R^{n}$.

**Definition 3.**  *Let $B\subseteq R^{n}$. A set A⊆B is said to be dense in B if, for every b∈B, there exists a sequence ${\left\{a_{i}\right\}}_{i=0}^{\infty }\subseteq A$ such that*

(114) $$\lim_{i\to \infty }a_{i}=b.$$

**Definition 4.**  *Let $B\subseteq R^{n}$. A set A⊆B is called nowhere dense in B if, for every open set U⊆B, A∩U is not dense in U.*

**Theorem 5.**  *Suppose that either*

*1.*  *$A⊊R^{n}$ is compact, or*
*2.*  *$A=R^{n}$, with k≠1.*

Then, $supp(F^{*})$ is nowhere dense in $R^{n}$.

**Proof.**  By Theorem 1, either $supp\left(F^{*}\right)=\left\{x_{0}\right\}$ for some $x_{0}\in R^{n}$ or

(115) $$Z\left(s;F^{*}\right)⊊R^{n}.$$

Since, in the former, case $supp(F^{*})$ is nowhere dense in $R^{n}$, assume the latter and let (115) hold. Let $U\subseteq R^{n}$ be a non-empty open set; we will show the result by proving that $Z(s;F^{*})\cap U$ is not dense in *U*.

Fix x∈U. Translating $s(\cdot ;F^{*})$ by x, rotating the coordinate system and applying Theorem 2 shows that there exists a sufficiently small open set *Q* containing x on which

(116) $$\begin{array}{cc} Z(s;F^{*})\cap Q & =\overset{n-1}{\underset{i=0}{⋃}}V_{i} \end{array}$$

(117) $$\begin{array}{cc} \; & =V_{0}\cup (\overset{n-1}{\underset{i=1}{⋃}}\overset{n_{i}}{\underset{j=0}{⋃}}\Gamma_{i}^{j}). \end{array}$$

It suffices to show the existence of a point of the form $(x^{n-1},u_{n})\in U\cap Q$ that is not the limit of any sequence in $Z(s;F^{*})\cap U\cap Q$. Let $\left(y_{m}^{n-1},y_{n,m}\right)$ be a convergent sequence in $Z(s;F^{*})\cap U\cap Q$, indexed by *m*, for which

(118) $$\begin{array}{c} \lim_{m\to \infty }y_{m}^{n-1}=x^{n-1}. \end{array}$$

Using the parameterization from (100), the *n*’th component of sequence index *m* satisfies one of the following:

1. $y_{n,m}\in \left\{v_{n}|v\in V_{0}\right\}$, or
2. for some $i_{m}\in \left\{1,\ldots ,n-1\right\}$ and $j_{m}\in \left\{1,\ldots ,n_{i_{m}}\right\}$,
  (119) $$y_{n,m}=\alpha_{i_{m}}^{j_{m},n}\left(y_{m}^{i_{m}}\right).$$
  Since each $\alpha_{i_{m}}^{j_{m},n}\left(\cdot \right)$ is real-analytic, it is continuous. Then, for $y_{n,m}$ satisfying (119), if $\lim_{m\to \infty }y_{n,m}$ exists, we have $\lim_{m\to \infty }y_{n,m}=\alpha_{i}^{j,n}\left(x^{i}\right)$ for some i∈{1,…,n−1} and $j\in \{1,\ldots ,n_{i}\}$. Since $V_{0}$ is either empty or a single point, the number of possible values for $\lim_{m\to \infty }{\left(y_{n}\right)}_{m}$ is at most
  (120) $$\begin{array}{cc} |V_{0}|\;+\sum_{i=1}^{n-1}n_{i} & <\infty . \end{array}$$

However, since x∈U∩Q, where U∩Q is open, the set $\left\{t\in R|\left(x^{n-1},x_{n}+t\right)\in U\cap Q\right\}$ is uncountable. Thus, there exists *t* such that $(x^{n-1},x_{n}+t)\in U\cap Q$ is not the limit of any sequence in $Z(s;F^{*})\cap U\cap Q$. □

## 4. Discussion

This paper has considered vector-valued channels with additive Gaussian noise. Unlike much of the prior work in this area, the noise was not limited to having independent and identically distributed components. The support of the capacity-achieving input distribution was discussed when inputs were subjected to an even-moment radial constraint of order 2k. Furthermore, the inputs were either allowed to take any value in $R^{n}$ or restricted to a compact set. When the input alphabet was the entire space, $R^{n}$, only the case k≥2 was considered since, for k=1, the optimal input distribution is well-known to be Gaussian.

The problem was framed as a convex optimization problem that was shown to be solved by a unique input distribution $F^{*}$. The conditions for optimality yielded a real-analytic function $s(\cdot ;F^{*})$ whose zero set contained $supp(F^{*})$, the support of $F^{*}$. Using the framework of an $L^{2}$ space that was weighted by the noise density, $s(\cdot ;F^{*})$ was simplified and shown to be non-constant on $R^{n}$. Through geometric analysis of the zero set of $s(\cdot ;F^{*})$, $supp(F^{*})$ was shown to be contained in a countable union of single points and submanifolds of dimensions ranging 1,…,n−1. When the input alphabet was compact, this union was further shown to be finite. Finally, it was determined that $supp(F^{*})$ has Lebesgue measure 0 and is nowhere dense in $R^{n}$.

This paper is an expansion of the work concerning even-moment input constraints in [14] to vector-valued channels that are not necessarily spherically symmetric. Viewed as a generalization of [12], it considers order 2k rather than second-moment radial constraints and includes $R^{n}$ as a possible input alphabet. Unlike prior work, it also provides geometric results on the supports of capacity-achieving inputs to spherically asymmetric channels.

## Data Availability

Not applicable.

## Appendix A. Convexity and Compactness of Optimization Space

**Theorem A1.** 
*The following properties hold for sets defined in Section 3.1:*

- *$F_{n}\left(A\right)$ is convex;*
- *for any b>0, $P_{n}\left(A,k,b\right)$ is convex and compact;*
- *$Q_{n}\left(A,k,a\right)$ is convex.*

**Proof.** We first show the convexity of $F_{n}\left(A\right)$. Let $F_{1},F_{2}\in F_{n}\left(A\right)$, t∈[0,1], and $F_{t}=tF_{1}+\left(1-t\right)F_{2}$. Then, since

(A1) $$supp\left(F_{t}\right)\subseteq supp\left(F_{1}\right)\cup supp\left(F_{2}\right)\subseteq A,$$

$F_{n}\left(A\right)$ is convex. To show convexity of $P_{n}\left(A,k,b\right)$ for b>0, let $F_{1},F_{2}\in P_{n}\left(A,k,b\right)$, t∈[0,1] and $F_{t}=tF_{1}+\left(1-t\right)F_{2}$. Since $P_{n}\left(A,k,b\right)\subseteq F_{n}\left(A\right)$, it suffices to show that $F_{t}$ satisfies the radial even-moment constraint:

(A2) $$\begin{array}{cc} \int_{R^{n}}{∥x∥}^{2k}\;dF_{t}\left(x\right) & =t\int_{R^{n}}{∥x∥}^{2k}\;dF_{1}\left(x\right)+\left(1-t\right)\int_{R^{n}}{∥x∥}^{2k}\;dF_{2}\left(x\right) \end{array}$$

(A3) $$\begin{array}{cc} \; & \leq tb+(1-t)b=b. \end{array}$$

We now show the convexity of $Q_{n}\left(A,k,a\right)$. For any $F_{1},F_{2}\in Q_{n}\left(A,k,a\right)$, there exists $b_{F_{1},F_{2}}>0$ for which $F_{1},F_{2}\in P\left(A,k,b_{F_{1},F_{2}}\right)$. Since $P(A,k,b_{F_{1},F_{2}})$ is convex, $tF_{1}+\left(1-t\right)F_{2}\in P\left(A,k,b_{F_{1},F_{2}}\right)\subseteq Q_{n}\left(A,k,a\right)$. Hence, $Q_{n}\left(A,k,a\right)$ is convex. It remains to show the compactness of $P_{n}\left(A,k,b\right)$ for any b>0. Note that the Lévy–Prokhorov metric metrizes weak convergence in $F(R^{n})$ [25]; so, sequential compactness is equivalent to compactness. To prove compactness of $P_{n}\left(A,k,b\right)$, we first show relative compactness, which allows us to conclude that any sequence in $P_{n}\left(A,k,b\right)$ has a subsequence that converges to some $F\in F_{n}\left(A\right)$. Further, showing that $F\in P_{n}\left(A,k,b\right)$ will complete the proof. Observe that each $F\in F(R^{n})$ is defined on the complete separable metric space $R^{n}$ equipped with Euclidean distance. By Prokhorov’s Theorem (Theorem 3.2.1 of [25]), the relative compactness of $P_{n}\left(A,k,b\right)$ is equivalent to the tightness of $P_{n}\left(A,k,b\right)$; so, we will prove the latter. To show the tightness of $P_{n}\left(A,k,b\right)$, let $X∼F\in P_{n}\left(A,k,b\right)$, ϵ>0, and $D={\left(a/\epsilon \right)}^{1/2k}$. Then, applying Markov’s inequality,

(A4) $$\begin{array}{cc} P\{X\in R^{n}\setminus \bar{B_{D}}\left(0\right)\} & =P\{∥X∥>D\} \end{array}$$

(A5) $$\begin{array}{cc} \; & \leq P\{∥X{∥}^{2k}\geq D^{2k}\} \end{array}$$

(A6) $$\begin{array}{cc} \; & \leq \frac{E[∥X{∥}^{2k}]}{D^{2k}} \end{array}$$

(A7) $$\begin{array}{cc} \; & \leq \frac{a}{D^{2k}} \end{array}$$

(A8) $$\begin{array}{cc} \; & =\epsilon . \end{array}$$

This is a uniform upper-bound for $F\in P_{n}\left(A,k,b\right)$; so, $P_{n}\left(A,k,b\right)$ is tight and, thus, relatively compact. By the relative compactness of $P_{n}\left(A,k,b\right)$, any sequence ${\left\{F_{m}\right\}}_{m=0}^{\infty }\subseteq P_{n}\left(A,k,b\right)$ has a subsequence ${\left\{F_{m_{j}}\right\}}_{m_{j}=0}^{\infty }$ that converges weakly to some $F\in F(R^{n})$. To show compactness, we must show that $F\in P_{n}\left(A,k,b\right)$. Since each $F_{m_{j}}\in P_{n}\left(A,k,b\right)$, it follows that

(A9) $$\int_{A}{∥x∥}^{2k}\;dF_{m_{j}}\left(x\right)\leq a.$$

By Theorem A.3.12 of [26], since ${∥x∥}^{2k}$ is non-negative and lower semicontinuous,

(A10) $$0\leq \int_{A}{∥x∥}^{2k}\;dF(x)\leq \underset{j\to \infty }{lim inf}\int_{A}{∥x∥}^{2k}\;dF_{m_{j}}\left(x\right)\leq a.$$

Therefore, the limiting distribution *F* satisfies the radial even-moment constraint imposed by $P_{n}\left(A,k,b\right)$. When $A=R^{n}$, we conclude that $F\in P_{n}\left(A,k,b\right)$. However, for the case that A is compact, we must also show almost surely that X∈A. For any index of the subsequence $m_{j}$,

(A11) $$\int_{A}\;dF_{m_{j}}\left(x\right)=1.$$

By the Portmanteau Theorem [27], since A is closed,

(A12) $$\int_{A}\;dF(x)\geq \underset{j\to \infty }{lim sup}\int_{A}\;dF_{m_{j}}\left(x\right)=1.$$

□

## Appendix B. Necessary Conditions for the Capacity-Achieving Distribution

**Theorem A2.** 
*Recall that*

(A13)
$$\begin{array}{cc} Q_{n}\left(A,k,a\right) & =\underset{b\geq a}{⋃}P_{n}\left(A,k,b\right). \end{array}$$

*Suppose that $F^{*}$ solves the optimization problem in (26), where γ≥0 is the Lagrange multiplier corresponding to the problem in (11). Then, the following are equivalent:*

P.1 *For every $F\in Q_{n}\left(A,k,a\right)$,*
  (A14)
  $$\int_{A}h\left(x;F^{*}\right)\;dF(x)\leq \gamma (\int_{A}{∥x∥}^{2k}\;dF(x)-a)+C+h\left(N\right).$$
P.2 *For all x∈A,*
  (A15)
  $$h\left(x;F^{*}\right)\leq {\gamma (∥x∥}^{2k}-a)+C+h\left(N\right),$$
  *and if $x\in supp(F^{*})$, then*
  (A16)
  $$h\left(x;F^{*}\right)={\gamma (∥x∥}^{2k}-a)+C+h\left(N\right).$$

**Proof.** For any $F\in Q_{n}\left(A,k,a\right)$, integrating both sides of (A15) with respect to dF(·) yields that (**P.2**) implies (**P.1**). It remains to be shown that (**P.1**) implies (**P.2**). Suppose this implication is false—that is, (**P.1**) holds but there either exists v∈A for which

(A17) $$h\left(v;F^{*}\right){>\gamma (∥v∥}^{2k}-a)+C+h\left(N\right),$$

or there exists $w\in supp(F^{*})$ such that

(A18) $$h\left(w;F^{*}\right)\neq {\gamma (∥w∥}^{2k}-a)+C+h\left(N\right).$$

If (A17) holds, let $b=\max\{∥v{∥}^{2k},a\}$ and let $F\left(x\right)=\prod_{i=1}^{n}u_{1}\left(x_{i}-v_{i}\right)$, where $u_{1}\left(\cdot \right)$ is the Heaviside step function. Then, $F\in P_{n}\left(A,k,b\right)\subseteq Q_{n}\left(A,k,a\right)$ and

(A19) $$\begin{array}{cc} \int_{A}h\left(x;F^{*}\right)\;dF(x)-\gamma (\int_{A}{∥x∥}^{2k}\;dF(x)-a) & =h\left(v;F^{*}\right)-{\gamma (∥v∥}^{2k}-a) \end{array}$$

(A20) $$\begin{array}{cc} \; & >C+h(N), \end{array}$$

contradicting (A14). Therefore, (A17) cannot be satisfied for any x∈A and we are left with the alternative that there exists $w\in supp(F^{*})\subseteq A$ for which (A18) hold—that is,

(A21) $$h\left(w;F^{*}\right){<\gamma (∥w∥}^{2k}-a)+C+h\left(N\right).$$

By Lemma A7, the extension of h(·;F) to $C^{n}$ is continuous; hence, h(·;F) is continuous on $R^{n}$. Since ${∥\cdot ∥}^{2k}$ is continuous as well, there exists δ>0 such that

(A22) $$h\left(x;F^{*}\right){<\gamma (∥x∥}^{2k}-a)+C+h\left(N\right)$$

for every $x\in B_{\delta }\left(w\right)$. Furthermore, since $w\in supp(F^{*})$, there exists ϵ such that $P\{X^{*}\in B_{\delta }\left(w\right)\}=\epsilon >0$. Recall from (18) that

(A23) $$h_{Y}\left(F^{*}\right)=-\int_{A}h\left(x;F^{*}\right)\;dF^{*}\left(x\right),$$

and since $\gamma g_{k}\left(F^{*}\right)=0$ (see (27)),

(A24) $$\gamma a=\gamma \int_{A}{∥x∥}^{2k}\;dF^{*}\left(x\right).$$

Substituting (A23) and (A24), and noting that $F^{*}\left(B_{\delta }\left(w\right)\cap A^{C}\right)=0$, yields

(A25) $$\begin{array}{cc} C+h(N)-\gamma a & =h_{Y}\left(F^{*}\right)-\gamma a \end{array}$$

(A26) $$\begin{array}{cc} \; & =\int_{A}[h\left(x;F^{*}\right)-\gamma {∥x∥}^{2k}]\;dF^{*}\left(x\right) \end{array}$$

(A27) $$\begin{array}{cc} \; & =\int_{A\cup B_{\delta }\left(w\right)}[h\left(x;F^{*}\right)-\gamma {∥x∥}^{2k}]\;dF^{*}\left(x\right) \end{array}$$

(A28) $$\begin{array}{cc} \; & =\int_{B_{\delta }\left(w\right)}[h\left(x;F^{*}\right)-\gamma {∥x∥}^{2k}]\;dF^{*}\left(x\right)\\ & \;+\int_{A\setminus B_{\delta }\left(w\right)}[h\left(x;F^{*}\right)-\gamma {∥x∥}^{2k}]\;dF^{*}\left(x\right) \end{array}$$

(A29) $$\begin{array}{cc} \; & <\epsilon [C+h(N)-\gamma a]+(1-\epsilon )[C+h(N)-\gamma a] \end{array}$$

(A30) $$\begin{array}{cc} \; & =C+h(N)-\gamma a, \end{array}$$

where the first term of (A29) is due to (A22) and the second is due to the contradiction derived from (A17). The above is a contradiction, which completes the proof. □

## Appendix C. Integrability Results

**Lemma A1.** 
*Let b>0 and $F\in P_{n}\left(R^{n},k,b\right)$. Then, there exist positive constants η and κ for which*

(A31)
$$\left|\ln p(y;F)\right|\leq {\eta ∥y∥}^{2}+\kappa$$

*for any $y\in R^{n}$.*

**Proof.** With $\eta =\frac{2}{\sigma_{1}^{2}}$, $D={\left(2b\right)}^{\frac{1}{2k}}$, and

(A32) $$M=\frac{1}{2\sqrt{{\left(2\pi \right)}^{n}\left|\Sigma \right|}},$$

let

(A33) $$\zeta_{y}=Me^{-\eta \max\{∥y{∥}^{2},D^{2}\}}.$$

Then, for any $y\in R^{n}$, $0<\zeta_{y}\leq p\left(y;F\right)\leq 2M$. Since |ln(·)| is continuous and has no local maxima,

(A34) $$\begin{array}{cc} |\ln p(y;F)|\; & \leq \max\left\{|\ln\left(\zeta_{y}\right)|,|\ln\left(2M\right)|\right\} \end{array}$$

(A35) $$\begin{array}{cc} \; & \leq \eta \max\{∥y{∥}^{2},D^{2}\}+|\ln M|+|\ln\left(2M\right)| \end{array}$$

(A36) $$\begin{array}{cc} \; & \leq {\eta ∥y∥}^{2}+\eta D^{2}+\left|\ln M\right|+|\ln\left(2M\right)|. \end{array}$$

□

**Lemma A2.** 
*For any $F\in P_{n}\left(R^{n},k,a\right)$, $\ln p(\cdot ;F)\in L_{p_{N}}^{2}\left(R^{n}\right)$*

**Proof.** The result follows by Lemma A1. □

**Lemma A3.** 
*Let β≥α≥1 and Z be a random variable taking values in $R^{n}$. If $E[∥Z{∥}^{\beta }]=\rho$, then $E[∥Z{∥}^{\alpha }]\leq \rho$ +1.*

**Proof.** Since β≥α≥1, we have

(A37) $$\begin{array}{cc} E[∥Z{∥}^{\alpha }] & \leq {E[\max\{1,∥Z∥\}}^{\alpha }] \end{array}$$

(A38) $$\begin{array}{cc} \; & \leq {E[\max\{1,∥Z∥\}}^{\beta }] \end{array}$$

(A39) $$\begin{array}{cc} \; & ={E[\max\{1,∥Z∥}^{\beta }\}] \end{array}$$

(A40) $$\begin{array}{cc} \; & \leq \rho +1. \end{array}$$

□

**Lemma A4.** 
*For any b>0 and $F_{0},F_{1}\in P_{n}\left(R^{n},k,b\right)$,*

(A41)
$$\begin{array}{cc} \int_{R^{n}}\left|p\left(y;F_{0}\right)\ln p(y;F_{1})\right|\;dy\; & <\infty . \end{array}$$

**Proof.** Note that

(A42) $$\begin{array}{cc} \int_{R^{n}}\left|p\left(y;F_{0}\right)\ln p(y;F_{1})\right|\;dy & =\int_{R^{n}}p\left(y;F_{0}\right)\left|\ln p(y;F_{1})\right|\;dy \end{array}$$

(A43) $$\begin{array}{cc} \; & =\int_{R^{n}}\int_{R^{n}}p_{N}\left(y-x\right)\left|\ln p(y;F_{1})\right|\;dF_{0}\left(x\right)\;dy. \end{array}$$

We proceed by first proving that

(A44) $$\begin{array}{cc} \int_{R^{n}}\int_{R^{n}}p_{N}\left(y-x\right)\left|\ln p(y;F_{1})\right|\;dy\;dF_{0}\left(x\right) & <\infty . \end{array}$$

Then, since the integrand is non-negative, the Tonelli–Fubini Theorem [18] justifies interchanging the order of integration in (A44) to conclude that (A43) and, hence, the left side of (A42) is finite. By Lemma A1, for any $y\in R^{n}$,

(A45) $$|\ln p(y;F_{1}){|\leq \eta ∥y∥}^{2}+\kappa_{b}.$$

Initially considering only the inner integral in (A44), with the substitution u=y−x, yields

(A46) $$\begin{array}{cc} \int_{R^{n}}p_{N}\left(y-x\right)\left|\ln p(y;F_{1})\right|\;dy & \leq \eta \int_{R^{n}}p_{N}\left(y-x\right){∥y∥}^{2}\;dy+\kappa_{b} \end{array}$$

(A47) $$\begin{array}{cc} \; & =\eta \int_{R^{n}}p_{N}\left(u\right){∥u+x∥}^{2}\;du+\kappa_{b} \end{array}$$

(A48) $$\begin{array}{cc} \; & \leq \eta \int_{R^{n}}p_{N}\left(u\right)(∥u∥+{∥x∥)}^{2}\;du+\kappa_{b} \end{array}$$

(A49) $$\begin{array}{cc} \; & \leq \eta \int_{R^{n}}p_{N}{\left(u\right)(4∥u∥}^{2}+{4∥x∥}^{2})\;du+\kappa_{b} \end{array}$$

(A50) $$\begin{array}{cc} \; & \leq {8\eta E[∥N∥}^{2}{]+8\eta ∥x∥}^{2}+\kappa_{b}, \end{array}$$

where (A48) is due to the triangle inequality. Since $E[∥N{∥}^{2}]=tr\left(\Sigma \right)$ is finite, there are positive constants

(A51) $$\begin{array}{cc} \kappa_{0} & ={8\eta E[∥N∥}^{2}]+\kappa_{b},and \end{array}$$

(A52) $$\begin{array}{cc} \;\kappa_{2} & =8\eta , \end{array}$$

for which

(A53) $$\int_{R^{n}}p_{N}\left(y-x\right)|\ln p(y;F_{1})|\;dy\leq \kappa_{2}{∥x∥}^{2}+\kappa_{0}.$$

Furthermore, since k≥1 and $E_{F_{0}}{[∥X∥}^{2k}]\leq b$, then by Lemma A3, $E_{F_{0}}{[∥X∥}^{2}]\leq b+1$. Substituting this and (A53) into (A44), we obtain

(A54) $$\begin{array}{cc} \int_{R^{n}}\int_{R^{n}}p_{N}\left(y-x\right)\left|\ln p(y;F_{1})\right|\;dy\;dF_{0}\left(x\right) & \leq \int_{R^{n}}(\kappa_{2}{∥x∥}^{2}+\kappa_{0})\;dF_{0}\left(x\right) \end{array}$$

(A55) $$\begin{array}{cc} \; & =\kappa_{2}E_{F_{0}}{[∥X∥}^{2}]+\kappa_{0} \end{array}$$

(A56) $$\begin{array}{cc} \; & \leq \left(b+1\right)\kappa_{2}+\kappa_{0} \end{array}$$

(A57) $$\begin{array}{cc} \; & <\infty . \end{array}$$

□

## Appendix D. Properties of the Objective Functional

The aim of this section is to discuss the weak continuity, strict concavity, and weak differentiability of the objective function,

(A58) $$J_{\gamma }\left(F\right)=I\left(F\right)-\gamma g_{k}\left(F\right),$$

for the optimization problem posed in (26). These properties are instrumental in the establishment and subsequent analysis of the convex optimization problem considered in Section 3. To support the proof of Theorem A2, we show that, for arbitrary b>0, the required properties hold on $P_{n}\left(A,k,b\right)$.

**Theorem A3.** 
*I(·) is weak continuous on $P_{n}\left(A,k,a\right)$.*

**Proof.** For any $F\in P_{n}\left(A,k,a\right)$, we write

(A59) $$\begin{array}{cc} I(F) & =h_{Y}\left(F\right)-h\left(N\right) \end{array}$$

(A60) $$\begin{array}{cc} \; & =h_{Y}\left(F\right)-\frac{1}{2}\ln\left|2\pi e\Sigma \right|, \end{array}$$

where $h_{Y}\left(F\right)$ is finite by Lemma A4. Therefore, weak continuity of I(·) on $P_{n}\left(A,k,a\right)$ is equivalent to weak continuity of $h_{Y}\left(\cdot \right)$ on $P_{n}\left(A,k,a\right)$. Let ${\left\{F_{m}\right\}}_{m=0}^{\infty }$ be a sequence in $P_{n}\left(A,k,a\right)$ converging weakly to $F\in P_{n}\left(A,k,a\right)$. By the Helly–Bray Theorem, since $p_{N}\left(\cdot \right)$ is bounded and continuous,

(A61) $$\begin{array}{cc} \lim_{m\to \infty }p\left(y;F_{m}\right) & =\lim_{m\to \infty }\int_{R^{n}}p_{N}\left(y-x\right)\;dF_{m}\left(x\right) \end{array}$$

(A62) $$\begin{array}{cc} \; & =\int_{R^{n}}p_{N}\left(y-x\right)\;dF(x) \end{array}$$

(A63) $$\begin{array}{cc} \; & =p(y;F), \end{array}$$

for any $y\in R^{n}$. Therefore, by Scheffé’s Lemma, the sequence ${\left\{p\left(y;F_{m}\right)\right\}}_{m=0}^{\infty }$ converges in total variation to p(y;F). It suffices to show that differential entropy is uniformly continuous over $\left\{p\left(\cdot ;\hat{F}\right)|\hat{F}\in P_{n}\left(A,k,a\right)\right\}$ with respect to the total variation metric. The family of densities $\left\{p\left(\cdot ;\hat{F}\right)|\hat{F}\in P_{n}\left(A,k,a\right)\right\}$ is uniformly upper bounded. Furthermore, the corresponding random vectors, Y=X+N for some $X∼\hat{F}\in P_{n}\left(A,k,a\right)$, uniformly satisfy the bound

(A64) E[∥Y∥]≤a+1+E[∥N∥]

given by Lemma A3. The result follows by Theorem 1 of [28]. □

**Theorem A4.** 
*For any b>0, I(·) is strictly concave on $P_{n}\left(A,k,b\right)$.*

**Proof.** See Appendix E of [14]. □

**Theorem A5.** 
*$g_{k}\left(\cdot \right)$ is convex on $Q_{n}\left(A,k,a\right)$.*

**Proof.** Let t∈[0,1] and $F_{0},F_{1}\in Q_{n}\left(A,k,a\right)$. Then, $g_{k}\left(F_{0}\right)$ and $g_{k}\left(F_{1}\right)$ are finite and

(A65) $$\begin{array}{cc} g_{k}\left(tF_{0}+\left(1-t\right)F_{1}\right) & =\int_{R^{n}}{∥x∥}^{2k}\;dtF_{0}\left(x\right)+\int_{R^{n}}{∥x∥}^{2k}\;d\left(1-t\right)F_{1}\left(x\right)-a \end{array}$$

(A66) $$\begin{array}{cc} \; & =t(\int_{R^{n}}{∥x∥}^{2k}\;dF_{0}\left(x\right)-a)+\left(1-t\right)(\int_{R^{n}}{∥x∥}^{2k}\;dF_{1}\left(x\right)-a) \end{array}$$

(A67) $$\begin{array}{cc} \; & =tg_{k}\left(F_{0}\right)+\left(1-t\right)g_{k}\left(F_{1}\right). \end{array}$$

□

We make use of the following notion of a derivative of a function defined on a convex set Ω [14].

**Definition A1.** 
*Define the weak derivative of L:Ω→R at $F_{0}$ in the direction F by*

(A68)
$$L^{\prime }\left(F_{0},F\right)=\lim_{t↓0}\frac{L\left(\left(1-t\right)F_{0}+tF\right)-L\left(F_{0}\right)}{t},$$

*whenever it exists.*

**Lemma A5.** 
*$g_{k}\left(\cdot \right)$ is weakly differentiable on $Q_{n}\left(A,k,a\right)$. For any $F_{0},F\in Q_{n}\left(A,k,a\right)$, the weak derivative is finite and given by*

(A69)
$$g_{k}^{\prime }\left(F_{0},F\right)=g_{k}\left(F\right)-g_{k}\left(F_{0}\right).$$

**Proof.** Let t∈[0,1] and $F_{0},F\in Q_{n}\left(A,k,a\right)$. Then, noting that $g_{k}\left(F_{0}\right)$ and $g_{k}\left(F_{1}\right)$ are finite,

(A70) $$\begin{array}{cc} g_{k}\left(tF+\left(1-t\right)F_{0}\right)-g_{k}\left(F_{0}\right) & =g_{k}\left(t\left(F-F_{0}\right)+F_{0}\right)-g_{k}\left(F_{0}\right) \end{array}$$

(A71) $$\begin{array}{cc} \; & \begin{array}{c} =\int_{A}{∥x∥}^{2k}\;d\left[t\left(F\left(x\right)-F_{0}\left(x\right)\right)+F_{0}\left(x\right)\right]-a\\ -(\int_{A}{∥x∥}^{2k}\;dF_{0}\left(x\right)-a) \end{array} \end{array}$$

(A72) $$\begin{array}{cc} \; & =t(\int_{A}{∥x∥}^{2k}\;dF\left(x\right)-\int_{A}{∥x∥}^{2k}\;dF_{0}\left(x\right)) \end{array}$$

(A73) $$\begin{array}{cc} \; & =t[\int_{A}{∥x∥}^{2k}\;dF\left(x\right)-a-(\int_{A}{∥x∥}^{2k}\;dF_{0}\left(x\right)-a)] \end{array}$$

(A74) $$\begin{array}{cc} \; & =t(g_{k}(F)-g_{k}(F_{0})). \end{array}$$

Dividing by *t* and taking the limit as it goes to 0 gives (A69). □

**Lemma A6.** 
*I(·) is weakly differentiable on $Q_{n}\left(A,k,a\right)$. For any $F_{0},F\in Q_{n}\left(A,k,a\right)$, the weak derivative at $F_{0}$ in the direction of F is given by*

(A75)
$$I^{\prime }\left(F_{0},F\right)=\int_{A}h\left(x;F_{0}\right)\;dF(x)-h_{Y}\left(F_{0}\right).$$

**Proof.** The proof largely follows Appendix E from [14]. The step that requires special attention is the application of the Dominated Convergence Theorem in (27) of [14]—that is, we would like to show the integrability of

(A76) $$\begin{array}{cc} |\left(p\left(y;F\right)-p\left(y;F_{0}\right)\right)\ln\left(\frac{1}{2}p\left(y;F_{0}\right)\right)|\; & \leq \;|p\left(y;F\right)\ln p(y;F_{0})\left|+\right|p\left(y;F_{0}\right)\ln p(y;F_{0})| \end{array}$$

(A77) $$\begin{array}{cc} \; & \;+(p\left(y;F\right)+p\left(y;F_{0}\right))\ln2. \end{array}$$

Since $F_{0},F\in Q_{n}\left(A,k,a\right)$, there exists b>0 for which $F_{0},F\in P_{n}\left(A,k,b\right)$. Then, (A77) follows by Lemma A4. □

## Appendix E. Analycity of Marginal Entropy Density

**Lemma A7.** 
*For any b>0 and $F\in P_{n}\left(A,k,b\right)$, the extension of h(x;F) to $z\in C^{n}$ given by*

(A78)
$$h\left(z;F\right)=-\int_{R^{n}}p_{N}\left(y-z\right)\ln p(y;F)\;dy$$

*is continuous in z.*

**Proof.** Let $z\in C^{n}$. Fix ϵ>0 and consider $\bar{B_{C^{n},\epsilon }}\left(z\right)$, the ball of radius ϵ around z in $C^{n}$. For any sequence ${\left\{z_{m}\right\}}_{m=0}^{\infty }\subseteq C^{n}$ converging to z, there exists M≥0 such that $z_{m}\in \bar{B_{C^{n},\epsilon }}\left(z\right)$ for each m≥M. Therefore, it suffices to show that $\lim_{m\to \infty }h\left(z_{m};F\right)=h\left(z;F\right)$ for each sequence ${\left\{z_{m}\right\}}_{m=0}^{\infty }\subseteq \bar{B_{C^{n},\epsilon }}\left(z\right)$. Since the extension of $p_{N}\left(u\right)$ to $C^{n}$ is continuous,

(A79) $$\begin{array}{cc} \lim_{m\to \infty }h\left(z_{m};F\right) & =\lim_{m\to \infty }-\int_{R^{n}}p_{N}\left(y-z_{m}\right)\ln p(y;F)\;dy \end{array}$$

(A80) $$\begin{array}{cc} \; & =-\int_{R^{n}}\lim_{m\to \infty }p_{N}\left(y-z_{m}\right)\ln p(y;F)\;dy \end{array}$$

(A81) $$\begin{array}{cc} \; & =-\int_{R^{n}}p_{N}\left(y-z\right)\ln p(y;F)\;dy \end{array}$$

(A82) $$\begin{array}{cc} \; & =h(z;F), \end{array}$$

where (A80) is due to the Dominated Convergence Theorem, which will be justified next. Let $y\in R^{n}$ and $z_{m}=\alpha_{m}+i\beta_{m}\in \bar{B_{C^{n},\epsilon }}\left(z\right)$. Prior to finding a dominating function for the entire integrand in (A80), we establish the following upper bound on $|p_{N}\left(y-z_{m}\right)|$:

(A83) $$\begin{array}{cc} |p_{N}\left(y-z_{m}\right)| & =\frac{1}{\sqrt{{\left(2\pi \right)}^{n}\left|\Sigma \right|}}\left|e^{-\frac{1}{2}\left(y-z_{m}\right)\Sigma^{-1}\left(y-z_{m}\right)}\right| \end{array}$$

(A84) $$\begin{array}{cc} \; & =\frac{1}{\sqrt{{\left(2\pi \right)}^{n}\left|\Sigma \right|}}e^{-\frac{1}{2}\left(y-\alpha_{m}\right)\Sigma^{-1}\left(y-\alpha_{m}\right)}e^{\frac{1}{2}\beta_{m}\Sigma^{-1}\beta_{m}} \end{array}$$

(A85) $$\begin{array}{cc} \; & \leq \frac{1}{\sqrt{{\left(2\pi \right)}^{n}\left|\Sigma \right|}}e^{-\frac{1}{2\sigma_{n}^{2}}{∥y-\alpha_{m}∥}^{2}}e^{\frac{1}{2\sigma_{1}^{2}}{∥\beta_{m}∥}^{2}} \end{array}$$

(A86) $$\begin{array}{cc} \; & \leq \frac{e^{\frac{1}{2\sigma_{1}^{2}}\epsilon^{2}}}{\sqrt{{\left(2\pi \right)}^{n}\left|\Sigma \right|}}e^{-\frac{1}{2\sigma_{n}^{2}}{∥y-\alpha_{m}∥}^{2}} \end{array}$$

(A87) $$\begin{array}{cc} \; & \leq \frac{e^{\frac{1}{2\sigma_{1}^{2}}\epsilon^{2}}}{\sqrt{{\left(2\pi \right)}^{n}\left|\Sigma \right|}}\max_{u\in \bar{B_{\epsilon }}\left(z\right)}e^{-\frac{1}{2\sigma_{n}^{2}}{∥y-u∥}^{2}} \end{array}$$

(A88) $$\begin{array}{cc} \; & \leq \frac{e^{\frac{1}{2\sigma_{1}^{2}}\epsilon^{2}}}{\sqrt{{\left(2\pi \right)}^{n}\left|\Sigma \right|}}\left\{\begin{array}{cc} e^{-\frac{1}{2\sigma_{n}^{2}}{\left(1-\frac{\epsilon }{∥y∥}\right)}^{2}{∥y∥}^{2}}, & \;\mathrm{if}\;y\notin \left\{0\right\}\cup \bar{B_{\epsilon }}\left(z\right)\\ 1, & \;\mathrm{if}\;y\in \left\{0\right\}\cup \bar{B_{\epsilon }}\left(z\right) \end{array}\right. \end{array}$$

(A89) $$\begin{array}{cc} \; & ≜\Theta (y). \end{array}$$

Now, by Lemma A4,

(A90) $$\begin{array}{cc} |p_{N}\left(y-z_{m}\right)\ln p(y;F)| & \leq \Theta \left(y\right)(\eta ∥y{∥}^{2}+\kappa_{b}), \end{array}$$

which is integrable with respect to y. □

**Lemma A8.** 
*For any $F\in P_{n}\left(A,k,a\right)$, h(x;F) has an analytic extension to an entire function on $C^{n}$.*

**Proof.** For convenience of notation, we will prove the case of n=2 here. Consider the extension of h(x;F) to $z\in C^{2}$:

(A91) $$\begin{array}{cc} h(z;F) & =-\int_{R^{2}}p_{N}\left(y-z\right)\ln p(y;F)\;dy \end{array}$$

(A92) $$\begin{array}{cc} \; & =-\int_{R^{2}}p_{N_{1}}\left(y_{1}-z_{1}\right)p_{N_{2}}\left(y_{2}-z_{2}\right)\ln p(y;F)\;dy \end{array}$$

(A93) $$\begin{array}{cc} \; & =-\int_{R^{2}}\kappa_{1}e^{-\frac{{\left(y_{1}-z_{1}\right)}^{2}}{2\sigma_{1}^{2}}}\kappa_{2}e^{-\frac{{\left(y_{2}-z_{2}\right)}^{2}}{2\sigma_{2}^{2}}}\ln p(y;F)\;dy, \end{array}$$

where, for i∈{1,2},

(A94) $$\kappa_{i}≜\frac{1}{\sqrt{2\pi \sigma_{i}^{2}}}.$$

We will show that $h(\left(z_{1},z_{2}\right);F)$ is an entire function in $z_{1}\in C$ for fixed $z_{2}$. Similarly, by the symmetry of the problem, $h(\left(z_{1},z_{2}\right);F)$ is an entire function in $z_{2}\in C$ for fixed $z_{1}$. We finally conclude, by Hartog’s Theorem [29], that $h(\left(z_{1},z_{2}\right);F)$ is entire on $C^{2}$. Therefore, it suffices to show that $h(\left(\cdot ,z_{2}\right);F)$ is entire, for which we use Morera’s Theorem. Morera’s Theorem requires that the function under consideration, in this case $h(\cdot ,z_{2};F)$, be continuous, which holds by Lemma A7. If, for any closed smooth curve θ(t), defined for 0≤t≤1, and any fixed $z_{2}\in C$,

(A95) $$\begin{array}{cc} \int_{0}^{1}h(\theta \left(t\right),z_{2};F)\;\theta^{\prime }\left(t\right)\;dt & =-\int_{0}^{1}\int_{-\infty }^{\infty }\int_{-\infty }^{\infty }\kappa_{1}e^{-\frac{{\left(y_{1}-\theta \left(t\right)\right)}^{2}}{2\sigma_{1}^{2}}}p_{N_{2}}\left(y_{2}-z_{2}\right)\times \\ & \;\;\ln p(\left(y_{1},y_{2}\right);F)d\;y_{1}d\;y_{2}\;\theta^{\prime }\left(t\right)\;dt \end{array}$$

(A96) $$\begin{array}{cc} \; & =0, \end{array}$$

then, by Morera’s Theorem, $h(\left(\cdot ,z_{2}\right);F)$ is entire. Furthermore, by the Fubini–Tonelli Theorem [18], if

(A97) $$\begin{array}{c} \int_{0}^{1}\int_{-\infty }^{\infty }\int_{-\infty }^{\infty }\left|\kappa_{1}e^{-\frac{{\left(y_{1}-\theta \left(t\right)\right)}^{2}}{2\sigma_{1}^{2}}}p_{N_{2}}\left(y_{2}-z_{2}\right)\ln p(\left(y_{1},y_{2}\right);F)\right|d\;y_{1}d\;y_{2}\;|\theta^{\prime }\left(t\right)|\;dt<\infty , \end{array}$$

then the order of integration in (A95) can be interchanged such that integration with respect to *t* is performed first. Under this condition, since the extension of

(A98) $$p_{N_{1}}\left(u_{1}\right)=\kappa_{1}e^{-\frac{u_{1}^{2}}{2\sigma_{1}^{2}}}$$

to $z_{1}\in C$ is analytic, we obtain

(A99) $$\int_{0}^{1}\kappa_{1}e^{-\frac{{\left(y_{1}-\theta \left(t\right)\right)}^{2}}{2\sigma_{1}^{2}}}p_{N_{2}}\left(y_{2}-z_{2}\right)\ln p(\left(y_{1},y_{2}\right);F)\;\theta^{\prime }\left(t\right)\;dt=0,$$

thereby fulfilling the condition for Morera’s Theorem in (A96). It remains only to justify the application of the Fubini–Tonelli theorem by showing (A97). To upper bound the integrand on the left side of (A97), let $\alpha_{0},\beta_{0}>0$ be sufficiently large such that

(A100) $$\left\{\theta \left(t\right)|0\leq t\leq 1\right\}\subseteq \left\{z\in C|Re\left\{z\right\}\in \left[-\alpha_{0},\alpha_{0}\right],Im\left\{z\right\}\in \left[-\beta_{0},\beta_{0}\right]\right\}.$$

By Lemma A1, there exists κ≥0 such that, for all $y\in R^{2}$,

(A101) $$\begin{array}{cc} \left|\ln p(y;F)\right| & \leq {\eta ∥y∥}^{2}+\kappa . \end{array}$$

We proceed by splitting the integral with respect to $y_{1}$ into two intervals:

(A102) $$\begin{array}{cc} A_{1} & ≜(-\infty ,0), \end{array}$$

(A103) $$\begin{array}{cc} A_{2} & ≜[0,\infty ). \end{array}$$

For 0≤t≤1, let θ(t)=α(t)+iβ(t). Let $y_{1}\in A_{2}$ and note that, since $y_{1}>0$,

(A104) $$\begin{array}{cc} -\frac{1}{2\sigma_{1}^{2}}{\left(y_{1}-\alpha \left(t\right)\right)}^{2} & \leq \frac{1}{2\sigma_{1}^{2}}\left(-y_{1}^{2}+2y_{1}\alpha_{0}-{\left(\alpha \left(t\right)\right)}^{2}\right) \end{array}$$

(A105) $$\begin{array}{cc} \; & \leq \frac{1}{2\sigma_{1}^{2}}\left(-y_{1}^{2}+2y_{1}\alpha_{0}\right) \end{array}$$

(A106) $$\begin{array}{cc} \; & =-\frac{1}{2\sigma_{1}^{2}}{\left(y_{1}-\alpha_{0}\right)}^{2}+\frac{1}{2\sigma_{1}^{2}}\alpha_{0}^{2}. \end{array}$$

From (A106), we obtain the upper bound

(A107) $$\begin{array}{cc} \left|e^{-\frac{{\left(y_{1}-\theta \left(t\right)\right)}^{2}}{2\sigma_{1}^{2}}}\right| & =\left|e^{-\frac{1}{2\sigma_{1}^{2}}\left({\left(y_{1}-\alpha \left(t\right)\right)}^{2}-{\left(\beta \left(t\right)\right)}^{2}-2i\left(y_{1}-\alpha \left(t\right)\right)\beta \left(t\right)\right)}\right| \end{array}$$

(A108) $$\begin{array}{cc} \; & =e^{\frac{1}{2\sigma_{1}^{2}}{\left(\beta \left(t\right)\right)}^{2}}e^{-\frac{1}{2\sigma_{1}^{2}}{\left(y_{1}-\alpha \left(t\right)\right)}^{2}} \end{array}$$

(A109) $$\begin{array}{cc} \; & \leq e^{\frac{1}{2\sigma_{1}^{2}}\left(\alpha_{0}^{2}+\beta_{0}^{2}\right)}e^{-\frac{1}{2\sigma_{1}^{2}}{\left(y_{1}-\alpha_{0}\right)}^{2}}. \end{array}$$

Therefore, substituting (A105), for any 0≤t≤1,

(A110) $$\begin{array}{cc} \; & \int_{0}^{\infty }\left|\kappa_{1}e^{-\frac{{\left(y_{1}-\theta \left(t\right)\right)}^{2}}{2\sigma_{1}^{2}}}\ln p(\left(y_{1},y_{2}\right);F)\right|\;dy_{1} \end{array}$$

(A111) $$\begin{array}{cc} & \;\leq \kappa_{1}e^{\frac{1}{2\sigma_{1}^{2}}\left(\alpha_{0}^{2}+\beta_{0}^{2}\right)}\int_{0}^{\infty }e^{-\frac{1}{2\sigma_{1}^{2}}{\left(y_{1}-\alpha_{0}\right)}^{2}}\left(\eta \left(y_{1}^{2}+y_{2}^{2}\right)+\kappa \right)\;dy_{1} \end{array}$$

(A112) $$\begin{array}{cc} \; & \;=c_{0}y_{2}^{2}+c_{1}, \end{array}$$

for some constants $0\leq c_{0},c_{1}<\infty$. Applying similar reasoning when $y_{1}\in A_{1}$ shows that there are constants $0\leq c_{2},c_{3}<\infty$ for which

(A113) $$\int_{-\infty }^{0}\left|\kappa_{1}e^{-\frac{{\left(y_{1}-\theta \left(t\right)\right)}^{2}}{2\sigma_{1}^{2}}}\ln p(\left(y_{1},y_{2}\right);F)\right|\;dy_{1}\leq c_{2}y_{2}^{2}+c_{3}.$$

We now integrate with respect to $y_{2}$. For any $z_{2}=\tau +ir\in C$ and 0≤t≤1,

(A114) $$\begin{array}{cc} \psi (\theta \left(t\right),z_{2}) & ≜\int_{-\infty }^{\infty }\int_{-\infty }^{\infty }\left|\kappa_{1}e^{-\frac{{\left(y_{1}-\theta \left(t\right)\right)}^{2}}{2\sigma_{1}^{2}}}p_{U_{2}}\left(y_{2}-z_{2}\right)\ln p(\left(y_{1},y_{2}\right);F)\right|d\;y_{1}d\;y_{2} \end{array}$$

(A115) $$\begin{array}{cc} \; & =\int_{-\infty }^{\infty }\sum_{i=1}^{2}\int_{A_{i}}\left|\kappa_{1}e^{-\frac{{\left(y_{1}-\theta \left(t\right)\right)}^{2}}{2\sigma_{1}^{2}}}\kappa_{2}e^{-\frac{{\left(y_{2}-z_{2}\right)}^{2}}{2\sigma_{2}^{2}}}\ln p(\left(y_{1},y_{2}\right);F)\right|d\;y_{1}d\;y_{2} \end{array}$$

(A116) $$\begin{array}{cc} \; & \leq \kappa_{2}e^{\frac{r^{2}}{2\sigma_{2}^{2}}}\int_{-\infty }^{\infty }e^{-\frac{{\left(y_{2}-\tau \right)}^{2}}{2\sigma_{2}^{2}}}[c_{0}y_{2}^{2}+c_{1}+c_{2}y_{2}^{2}+c_{3}]d\;y_{2} \end{array}$$

(A117) $$\begin{array}{cc} \; & <\infty . \end{array}$$

Therefore, $\psi (\theta \left(t\right),z_{2})$ is uniformly bounded over {θ(t)|0≤t≤1} and

(A118) $$\int_{0}^{1}\left|\psi \left(\theta \left(t\right),z_{2}\right)\;\theta^{\prime }\left(t\right)\right|\;dt<\infty .$$

□

## Appendix F. Polynomial Bounds

**Lemma A9.** 
*The function*

(A119)
$$f\left(L\right)=\min_{∥y∥=L}\sum_{i=1}^{n}y_{i}^{2k}$$

*satisfies $f\left(L\right)\geq L^{2k}/n^{k}$.*

**Proof.** First, note that, for any L≥0 and $y\in R^{n}$ satisfying L=∥y∥, we have

(A120) $$\begin{array}{cc} \max_{i\in \{1,\ldots ,n\}}y_{i}^{2} & \geq \frac{L^{2}}{n}. \end{array}$$

Then, for any L≥0, we obtain the lower bound

(A121) $$\begin{array}{cc} f(L) & =\min_{∥y∥=L}\sum_{i=1}^{n}y_{i}^{2k} \end{array}$$

(A122) $$\begin{array}{cc} \; & \geq \min_{∥y∥=L}\max_{i\in \{1,\ldots ,n\}}y_{i}^{2k} \end{array}$$

(A123) $$\begin{array}{cc} \; & \geq \frac{L^{2k}}{n^{k}}. \end{array}$$

□

**Lemma A10.** 
*For any $l\in Z_{\geq 0}^{n}$, the function*

(A124)
$$\xi_{l}\left(L\right)=\max_{∥y∥=L}\left|y^{l}\right|$$

*satisfies $\xi_{l}\left(L\right)\leq L^{d(l)}$, where $d\left(l\right)=l_{1}+\ldots +l_{n}$.*

**Proof.** For ∥y∥=L,

(A125) $$\begin{array}{cc} \xi_{l}\left(L\right) & =\max_{∥y∥=L}\left|y^{l}\right| \end{array}$$

(A126) $$\begin{array}{cc} \; & =\max_{∥y∥=L}\left|y_{1}^{l_{1}}\cdots y_{n}^{l_{n}}\right| \end{array}$$

(A127) $$\begin{array}{cc} \; & \leq \max_{∥y∥=L}L^{l_{1}}\cdots L^{l_{n}} \end{array}$$

(A128) $$\begin{array}{cc} \; & =L^{d(l)}. \end{array}$$

□
